# Cell differentiation controls iron assimilation in the choanoflagellate *Salpingoeca rosetta*

**DOI:** 10.1128/msphere.00917-24

**Published:** 2025-02-26

**Authors:** Fredrick Leon, Jesus M. Espinoza-Esparza, Vicki Deng, Maxwell C. Coyle, Sarah Espinoza, David S. Booth

**Affiliations:** 1 Department of Biochemistry and Biophysics, University of California, San Francisco School of Medicine, San Francisco, California, USA; 2 Tetrad Graduate Program, University of California, San Francisco, California, USA; 3 Department of Molecular and Cell Biology, Howard Hughes Medical Institute, University of California, Berkeley, California, USA; 4 Chan Zuckerberg Biohub SF, San Francisco, California, USA; NC State University, Raleigh, North Carolina, USA

**Keywords:** choanoflagellate, cell-type evolution, iron colloid, marine microeukaryote, cytochrome b561

## Abstract

Marine microeukaryotes have evolved diverse cellular features that link their life histories to surrounding environments. How those dynamic life histories intersect with the ecological functions of microeukaryotes remains a frontier to understanding their roles in critical biogeochemical cycles. Choanoflagellates, phagotrophs that cycle nutrients through filter feeding, provide models to explore this intersection, for many choanoflagellate species transition between life history stages by differentiating into distinct cell types. Here, we report that cell differentiation in the marine choanoflagellate *Salpingoeca rosetta* endows one of its cell types with the ability to utilize insoluble ferric colloids. These colloids are a predominant form of iron in marine environments and are largely inaccessible to cell-walled microbes. Therefore, choanoflagellates and other phagotrophic eukaryotes may serve critical ecological roles by cycling this essential nutrient through iron utilization pathways. We found that *S. rosetta* can utilize these ferric colloids via the expression of a cytochrome b561 iron reductase (*cytb561a*). This gene and its mammalian ortholog, the duodenal cytochrome b561 (*DCYTB*) that reduces ferric cations for uptake in gut epithelia, belong to a subgroup of cytochrome b561 proteins with distinct biochemical features that contribute to iron reduction activity. Overall, our findings provide insight into the ecological roles choanoflagellates perform and inform reconstructions of early animal evolution where functionally distinct cell types became an integrated whole at the origin of animal multicellularity.

**IMPORTANCE:**

This study examines how cell differentiation in a choanoflagellate enables the uptake of iron, an essential nutrient. Choanoflagellates are widespread, aquatic microeukaryotes that are the closest living relatives of animals. Similar to their animal relatives, we found that the model choanoflagellate, *S. rosetta*, divides metabolic functions between distinct cell types. One cell type uses an iron reductase to acquire ferric colloids, a key source of iron in the ocean. We also observed that *S. rosetta* has three variants of this reductase, each with distinct biochemical properties that likely lead to differences in how they reduce iron. These reductases are variably distributed across ocean regions, suggesting a role for choanoflagellates in cycling iron in marine environments.

## INTRODUCTION

Phagotrophic microeukaryotes support the flow of nutrients in their environments by feeding on bacteria, picophytoplankon (1), and viruses (2, 3). Phagotrophs can further breakdown essential nutrients that form insoluble precipitates (e.g., iron [4], phosphates [5], and polysaccharides [6, 7]), thereby liberating nutrients that would otherwise be inaccessible to phytoplankton (4, 8) and bacteria (9) that drive critical biogeochemical cycles (10, 11). Of the phagotrophs that support these nutrient cycles, choanoflagellates distinguish themselves as highly efficient filter feeders that are widely distributed in aquatic environments (12–14). Choanoflagellates can ingest an array of prey (1–3) and inorganic particles (15); however, it remains unknown how choanoflagellates—and most other phagotrophs—digest nutrients and release them into the surrounding environment. Investigating those molecular mechanisms has historically been hindered by the limited number of systems and genetic tools established for marine microeukaryotes (16, 17). Molecular insights would better establish the specific functions that phagotrophs perform in marine ecosystems, rather than ascribe those functions from potentially misleading genomic comparisons (18).

**Fig 1:**
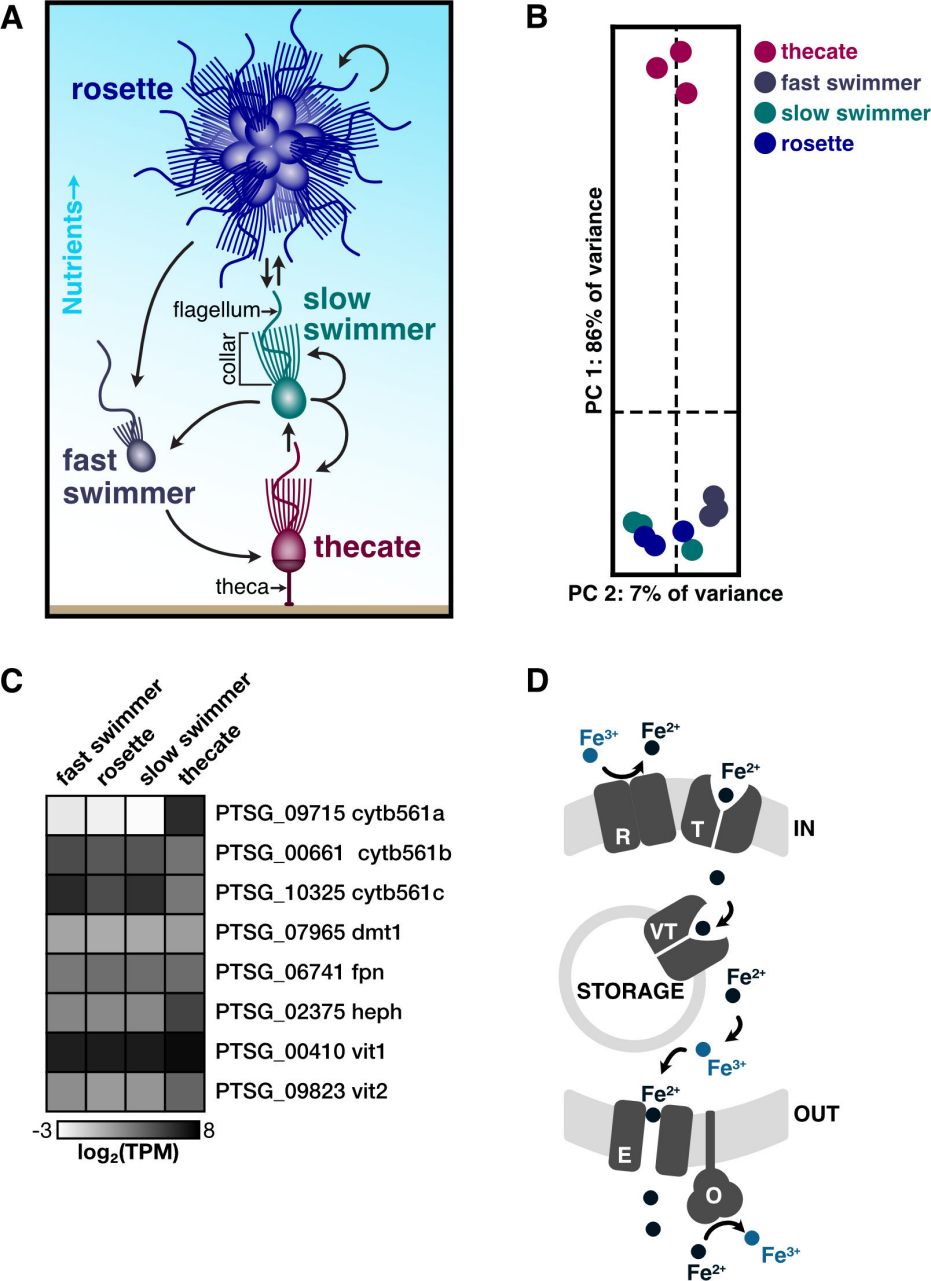
Thecates from the choanoflagellate *S. rosetta* are a distinct cell type that upregulates a cytochrome b561 reductase. (**A**) *S. rosetta* differentiates into morphologically distinct cell types. This schematic shows four of the cell types from *S. rosetta* that were stably cultured to produce transcriptome profiles. All of these cell types display the common choanoflagellate cell architecture in which an apical flagellum is encircled by a collar of actin-filled microvilli (indicated on the slow swimmer) that enables choanoflagellates to phagocytose bacteria and particulate matter. When nutrients are abundant, slow swimmers (green) respond to bacterial and algal cues to develop into multicellular rosettes (blue) that form by serial cell division. Cultures of slow swimmers also form chains of cells through serial cell divisions, but those chains are easily disrupted by mechanical force. Under starvation, slow swimmers and rosettes become fast swimmers (gray), which have a reduced cell body and collar with a longer flagellum. Under sustained nutrient deprivation, fast swimmers differentiate into thecates (red), a type of cell that adheres to substrates by constructing an extracellular apparatus called a theca. Thecates still proliferate in low nutrient conditions by dividing into swimmers that build their own theca. By taking the supernatant of thecate cultures in high nutrient conditions, thecates can then differentiate into slow swimmers. (**B**) The transcriptome profile of thecates stands apart from all other cell types. A principal component analysis of triplicate RNA-seq profiles from each cell type (panel A) shows that 86% of the variance between samples is attributed to the thecate transcriptome profile. All other cell types cluster closely together. (**C**) Cell-type expression of iron acquisition genes identified *S. rosetta* cell types. Genes are denoted by unique identifiers for *S. rosetta* genes and gene names that were given in this work based on their homology with animals. Expression values are averaged triplicate values of transcripts per million (TPM) from cell type transcriptomes (B). Of all genes and paralogs, *cytb561a* exhibits the most striking differential regulation. (**D**) Diagram of iron transport pathway based on identified iron acquisition homologs. We hypothesize one or more Cytb561 reductases (R) reduce ferric iron to ferrous iron to be trafficked across the cell membrane by ion transporter Dmt1 (T). Inside the cell, ferrous iron can be metabolized and utilized or stored in vacuoles by vacuolar transporters Vit1/2 (VT). Upon leaving the cell, ferrous iron is exported across the membrane by Fpn as an efflux pump (E] )and finally oxidized back to ferric iron by the oxidase Heph (O).

The environments where choanoflagellates may influence nutrient cycles depend on their life history. Choanoflagellates transition between different stages of their life history by differentiating into distinct cell types (12, 19, 20). In the emerging model choanoflagellate *Salpingoeca rosetta*, diverse environmental cues promote the transitions between different types of cells (21) (Fig. 1A). Under nutrient-replete conditions, sessile thecate cells differentiate into motile slow swimmers that can proliferate as single cells or chains of cells connected through intercellular bridges (21–23). In response to specific bacterial (24–26) and algal (27) cues, slow swimmers develop into rosettes, which are multicellular colonies that form through serial cell division (28). As rosettes or slow swimmers starve, they become fast swimmers, and under prolonged nutrient limitation, fast swimmers differentiate into thecates that can proliferate under similarly harsh conditions (21). The recapitulation of these cell type transitions in the laboratory makes *S. rosetta* a promising model to investigate how a marine microeukaryote integrates environmental signals to produce distinct cell types. However, the potential functions that *S. rosetta* cell types could perform in their environments remain to be uncovered, as choanoflagellate cell types have mostly been defined through their morphological features (19, 21, 28, 29) rather than molecular and metabolic functions.

Uncovering cell-type-specific function(s) in choanoflagellates promises to illuminate how specialized cell types evolved as multicellular development originated in animals, the closest living relatives of choanoflagellates. Cell differentiation is markedly different in animals compared with their unicellular relatives. In animals, cells differentiate from pluripotent progenitors into terminal cell types with specific functions in building and connecting complex tissues (30). In choanoflagellates, filasterea, and teretosporea—which, together with animals, form the Holozoan clade—cells reversibly differentiate to transition between life history stages and environmental contexts (21, 31–34), but there is little evidence of what role(s) those differentiated cell types perform in their environment. Cell-type-specific differences in metabolic functions could impact the surrounding environment where those microeukaryotes reside (35). Functional differences between the cell types of non-animal holozoans would further substantiate the hypothesis that animals co-opted functionally specific cell types to evolve their multicellular bodies (36–42).

We set out to identify the functional differences between choanoflagellate cell types by first refining the transcriptomes of rosettes, slow swimmers, thecates, and fast swimmers from *S. rosetta*. In doing so, we found one gene encoding a cytochrome b561 iron reductase that was highly expressed in the thecate cell type. An ortholog of this gene, the mammalian duodenal cytochrome b561 (*DCYTB*) regulates iron transport across the gut epithelium (43). By comparing the growth of thecates and slow swimmers in the presence of different sources of iron, we observed that thecates could ingest insoluble ferric colloids and assimilate iron from these particles for enhanced growth. With CRISPR/Cas9 genome editing, we established that the thecate-specific expression of a cytochrome b561 iron reductase is necessary for the improvement in growth. A phylogeny of cytochrome b561 homologs uncovered functional differences between cytochrome b561 proteins that may influence how different homologs reduce iron. The cytochrome b561 homologs have variable copy numbers across choanoflagellate lineages, and metatranscriptomic and metagenomic data from the open ocean revealed distinct distributions of gene abundance and expression. Overall, these results demonstrate at least one environmental function of a choanoflagellate controlled by cell differentiation. This function—the assimilation of iron from ferric colloids—further points to the importance of choanoflagellates in relieving nutrient limitations in open ocean environments by digesting insoluble, essential nutrients.

## RESULTS

### Thecates display a different transcriptome profile than other cell types

We refined previous *S. rosetta* transcriptomes (44) to uncover functional differences between cell types reflected in their gene expression profiles (Table S1). To improve those transcriptomes, we took advantage of methods to stably culture thecate, slow swimmer, rosette, and fast swimmer cell types (Fig. 1A) in monoxenic strains of *S. rosetta* that feed on the bacterium *Echinicola pacifica* (22, 23, 45). Slow swimmers, rosettes, and fast swimmers were derived from a common strain (PRA-390), whereas thecates were from another strain (HD1) that was isolated during the initial establishment of monoxenic cultures (22). We also developed a method to preferentially lyse *S. rosetta* while discarding their feeder bacteria to harvest mRNA from *S. rosetta* with a single round of poly-A selection (Fig. S1) rather than previous methods that relied on multiple rounds of selection to deplete contaminating RNAs from bacteria (44, 46). A principal component analysis of these improved transcriptomes emphasized the difference between the thecate cell type compared with slow swimmers, rosettes, and fast swimmers (Fig. 1B) with the first principal component accounting for 86% of the variance and clearly separating thecates from all other cell types. Although these results are consistent with previous gene expression profiles that found thecates separated from other types of cells (44, 47), the replicate measures in our data do not show a clear difference between slow swimmers, rosettes, and fast swimmers, suggesting that these three cell types are phenotypically plastic states.

The contrast between thecates and other cell types prompted us to investigate what unique functions thecates may perform. By comparing thecates to slow swimmers, we identified a set of genes that reproducibly (*q* < 0.01, note that *q* is a *P*-value adjusted for multiple comparisons) displayed a greater than 2-fold change in gene expression in thecates. We analyzed this set of genes for common gene ontologies (GO) (48–52) that indicated functional categories enriched in thecates, keeping in mind that GO analysis may underestimate functional differences between cell types due to incomplete genome annotations in this emerging model system (53). Genes upregulated in thecates were clearly enriched for functional modules (Fig. S2) implicated in cell signaling (e.g., nucleotide binding, protein kinase, protein phosphatase, calcium binding, metal ion transporter, guanylate cyclase, and G-protein coupled receptor), gene regulation (e.g., transcription factor and ribosome component), and metabolism (e.g., nucleotide binding, oxidoreductase, glycosyl hydrolase, metal ion transporter, carbohydrate binding, sulfuric ester hydrolase, and ascorbic acid binding). In contrast, swimmers expressed modules for cell division, RNA processing, and oxidative metabolism (Fig. S2). This comparative analysis highlighted a gene (PTSG_09715; Fig. 1C, row 1) with 502-fold higher expression in thecates compared with slow swimmers (*q* < 10^−40^) that was annotated as an oxidoreductase (GO:0016491) composed of a single cytochrome b561 iron reductase domain (Pfam 03188), leading us to rename this gene *cytb561a*.

To determine if other genes involved in iron metabolism may also be differentially regulated, we searched the genome of *S. rosetta* for homologs of well-annotated iron acquisition genes (Fig. 1C; https://doi.org/10.6084/m9.figshare.28225106). We found two additional paralogs of the cytochrome b561 reductase: *cytb561b,* which had 2.5-fold lower expression in thecates (*q* < 10^−3^), and *cytb561c,* which was not differentially expressed (*q* > 0.1). We further identified putative iron acquisition genes in *S. rosetta* based on the presence of protein domains that mediate iron transport in animals: *dmt1*, a homolog to the animal divalent metal transporter 1 (*DMT1*); *fpn,* a homolog to ferroportin (*FPN*), which is a ferrous efflux transporter; and *heph,* a homolog of the ferrous oxidase hephaestin (*HEPH*), which displayed 8-fold higher expression in thecates than slow swimmers (*q* < 10^−30^). Additionally, two paralogs of vacuolar iron transporters (*vit1* and *vit2*), which help store iron in the vacuoles of fungal cells (54, 55), were expressed in *S. rosetta*. Of those two genes, only the expression of *vit2* increased in thecates (4.4-fold, *q* < 10^−10^). Together, these homologs represent a putative module for iron import, storage, and export (Fig. 1D), in which reductases [R] reduce iron for transport across membranes by transporters [T], iron may be stored in vacuoles by vacuolar transporters [VT], and iron is exported by an efflux transporter [E] and oxidized by an oxidase [O]. Overall, the genes within this module have consistent expression across cell types, except for *cytb561a*. Therefore, we further investigated how *cytb561a* influences iron acquisition in thecates.

### *cytb561a* expression improves thecate cell growth with ferric colloids

**Fig 2:**
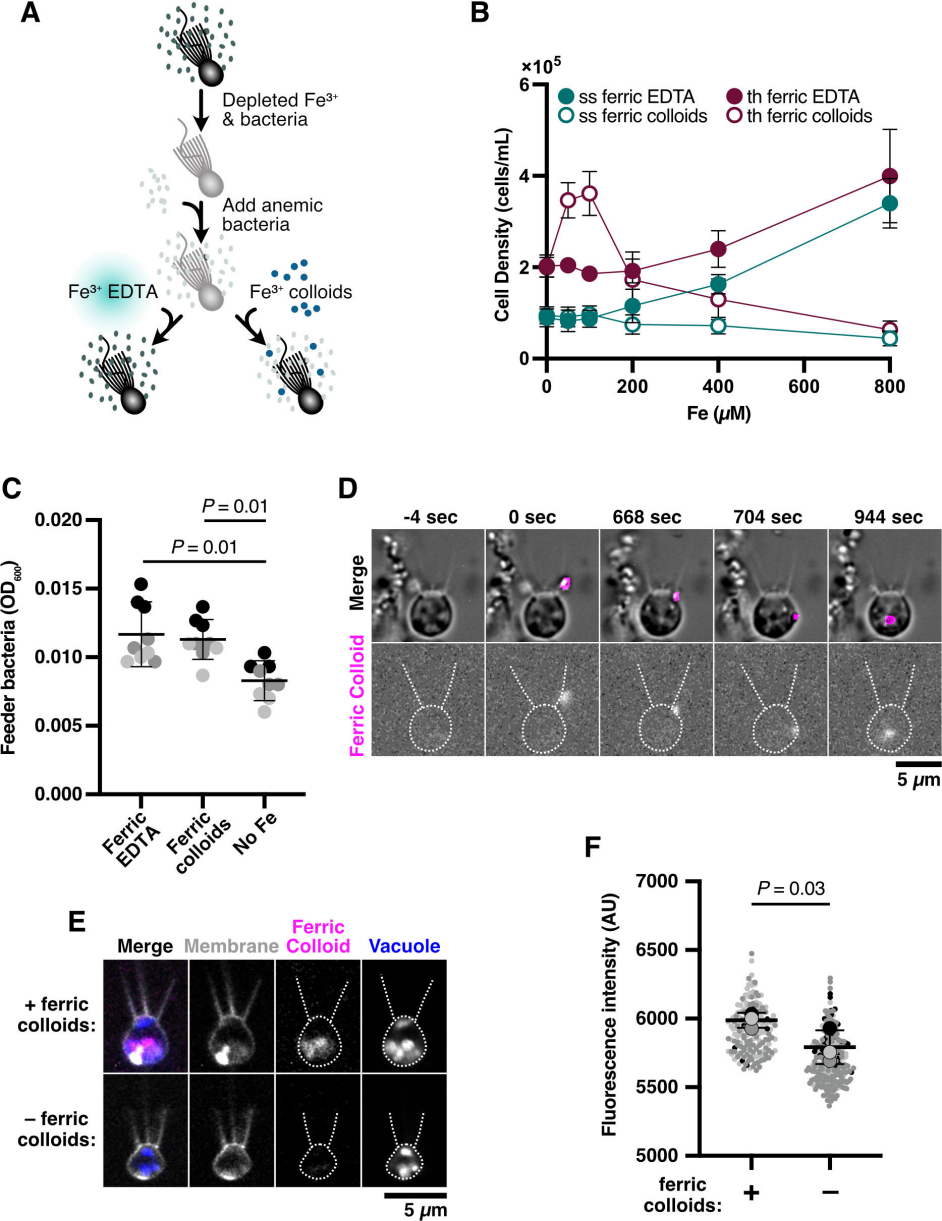
Thecates relieve iron limitation through the ingestion of ferric colloids. (**A**) A method to swap iron sources that support choanoflagellate growth. In preparation for iron limitation assays, *S. rosetta* (strain PRA-390) was passaged in low-nutrient media and depleted of iron. Cells were then washed multiple times and inoculated with iron-limited feeder bacteria. At the same time, ferric EDTA (Fe^3+^•EDTA) or ferric colloids (Fe^3+^) were provided. Cultures grew for 48 h at 27°C before assaying *cytb561a* growth (B) (Fig. 3B; Fig. S3) and expression (Fig. 3C; Fig. S7). (**B**) Thecates exhibit improved growth with low concentrations of ferric colloids compared with slow swimmers. The cell density of slow swimmer and thecate cultures when grown with titrations of ferric EDTA or ferric colloids. Ferric colloids at 50–100 μM ferric colloids supported higher cell densities for thecates. Meanwhile, higher concentrations of ferric EDTA (≥ 200 µM) supported higher growth in both cell types compared with ferric colloids. However, the cell density of cultures grown with 800 µM ferric EDTA did not exceed that of thecates grown with 100 µM ferric colloids. (**C**) *E. pacifica* grows similarly with ferric EDTA or ferric colloids. Because ferric colloids would influence OD600 measurements in a standard growth curve experiment, *E. pacifica* cultures were grown for 48 h with 100 µM ferric EDTA or ferric colloids and then lightly centrifuged at 500 × *g* for 1 min at room temperature to settle iron particulates. Afterward, the supernatant OD_600_ was measured. Gray scale represents matching replicates, the black bar denotes the global mean, and *P-*values were calculated using Tukey’s multiple comparisons. (**D**) Thecates ingest ferric colloids through phagocytosis. Time courses of wild-type thecates incubated with fluorescently labeled ferric colloids (see methods on ferric colloid labeling). Fluorescent ferric colloid particles were tracked (magenta outline) and observed being ingested and internalized through phagocytosis. The cell outline and feeding collar are marked by dotted lines. The zero-time point indicates initial contact with the ferric colloid particle. (**E**) Ingested ferric colloids reside within food vacuoles. Confocal microscopy of wild-type thecates incubated with and without fluorescently labeled ferric colloids. Split channels show membranes (gray), ferric colloids (magenta), and vacuoles (blue). The cell outline and feeding collar are marked by dotted lines. (**F**) Populations of thecates exhibit widespread ferric colloid ingestion. Fluorescence intensity of thecate cultures incubated with (+) and without (–) fluorescently labeled ferric colloids. Individual cell intensities from a replicate experiment (*N* = 3) are shaded in the same gray hue, and larger circles indicate the mean calculated by fitting a gamma distribution to a replicate population. *P-*values were calculated from the mean values with a one-tailed *t*-test.

Given the role that oxidoreductases perform in diverse eukaryotes to regulate iron acquisition (56–59), we hypothesized that the strong induction of *cytb561a* could enable thecates to reduce ferric ions from the environment for internalization through a divalent metal transporter. We first tested if thecate and slow swimmer cultures that were derived from the same initial strain (PRA-390) grew differently as the concentration and source of ferric ions were varied. Ferric ions were added to an iron-depleted media formulation (Fig. 2A; Table S2) as either soluble ferric EDTA (Fe^3+^•ethylenediaminetetraacetic acid) or as insoluble ferric colloids (Fe^3+^(oxyhydr)oxides) (60). Both forms of iron are ecologically important(61, 62), for models based on environmental sampling predict that 40% of the world’s surface ocean waters rely on colloidal sources of iron and 18% on soluble iron chelates (61).

**Fig 3:**
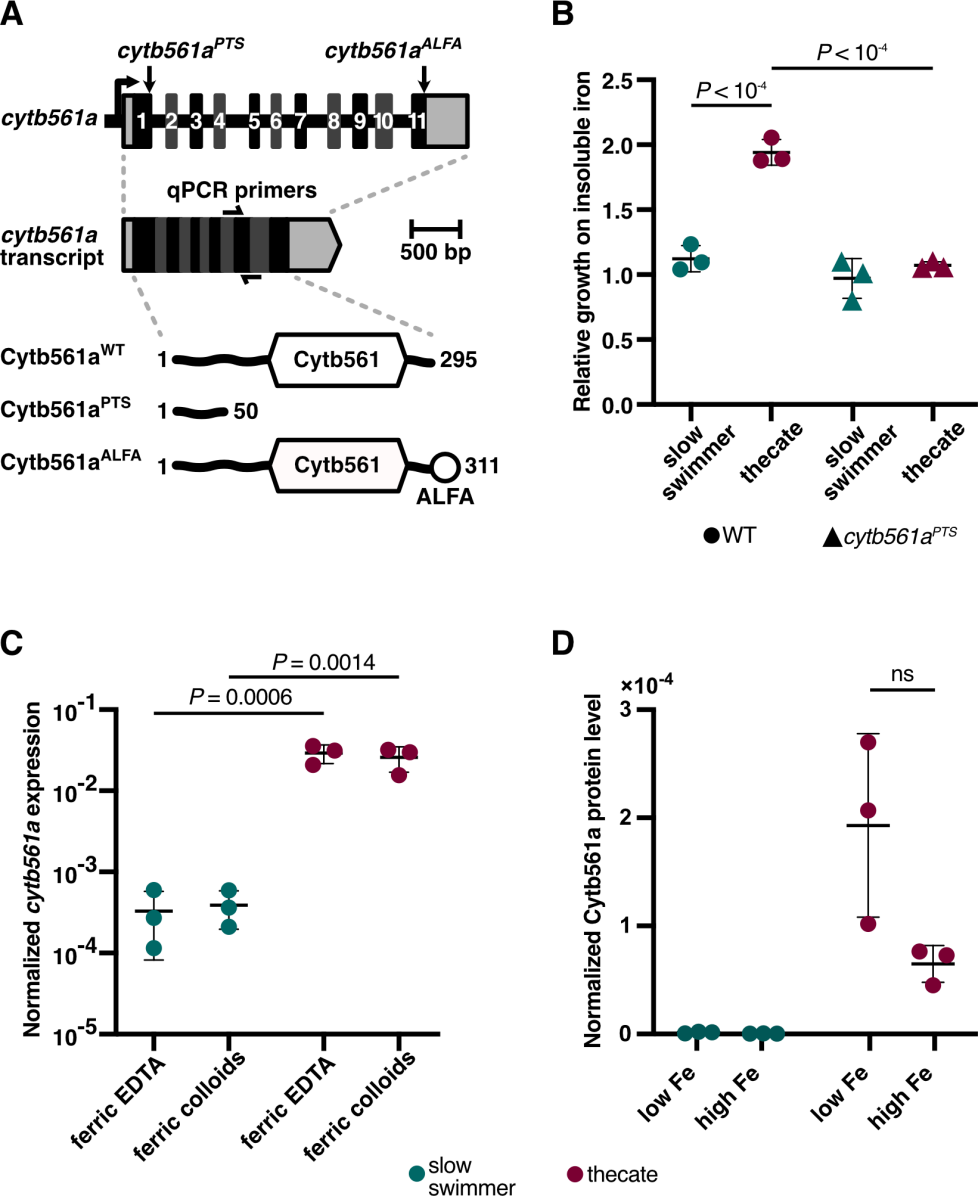
*cytb561a* expression is necessary for the utilization of ferric colloids. (**A**) The endogenous locus of *cytb561a* was edited to produce hypomorphic and epitope-tagged alleles. Diagram of endogenous genomic locus of *cytb561a* shows the position of premature terminal sequence (PTS) and epitope tag (ALFA-tag) insertions. The position of qPCR primers is shown as well, spanning exon-exon junctions. The predicted protein products of each insertion are shown as well, with the cytochrome b561 domain labeled as cytb561. (**B**) Thecates require *cytb561a* for increased proliferation with ferric colloids. To account for differences in growth between slow swimmers (green) and thecates (red), the cell density of cultures grown with ferric colloids was normalized to cultures grown with ferric EDTA. With this metric, a ratio greater than one indicates that the cell type displays increased growth with ferric colloids, whereas a ratio less than one indicates the converse. A premature termination sequence introduced at position 151 in *cytb561a* with CRISPR/Cas9 genome editing produced a mutant allele (*cytb561a^PTS^*) with a 3.6-fold reduction in *cytb561a* mRNA levels and stop codons that would truncate the protein translated from this transcript (Fig. S2; https://doi.org/10.6084/m9.figshare.28225106). Unlike the wild-type strain (circles), thecate cells with *cytb561a^PTS^* displayed no improved growth with ferric colloids (triangles). *P-*values were calculated from a two-way ANOVA. (**C**) *cytb561a* expression is part of the thecate regulon and not determined by external iron conditions. The expression of *cytb561a* was monitored by RT-qPCR in cultures of slow swimmers (green) or thecates (red) that were grown with either ferric EDTA or ferric colloids. The expression of *cytb561a* was normalized to *cofilin* (PTSG_01554)*,* a eukaryotic gene that displays high, consistent expression across all *S. rosetta* cell types. Independent triplicates were performed, and *P-*values were calculated from a two-way ANOVA. (**D**) Slow swimmers do not respond to iron availability at the protein level of Cytb561a. Cytb561a protein levels were detected using an endogenously edited epitope tag and normalized to total protein levels via western blot (raw images, Fig. S8). Proteins levels were measured from the cultures of slow swimmers (green) or thecates (red) that were grown with either low iron media (4% PG, 0.27 ± 0.03 µM Fe) or nutrient-replete media (25% RA, 11.7 µM added Fe). *P-*values were calculated from a two-tailed *t*-test.

Lower concentrations of ferric colloids stimulated the growth of thecates but not slow swimmers. Without any iron supplements, the baseline carrying capacity of thecates was higher than slow swimmers, indicating that thecates grow better in nutrient-limited conditions. When supplemented with 50 or 100 µM ferric colloids, thecates grew to a 2-fold higher cell density (Fig. 2B; Fig. S3A through C, Tukey’s multiple comparisons, *P* < 10^−4^ for 100 µM ferric colloids); however, slow swimmers showed no change at the same concentration (Tukey’s multiple comparisons, *P* > 0.1). Higher concentrations of ferric EDTA (≥200 µM) stimulated greater cell proliferation, but the same concentrations of ferric colloids did not. However, even at the highest concentrations of ferric EDTA, neither cell type exceeded the density of thecates supplemented with 100 µM ferric colloids (Tukey’s multiple comparisons, *P* > 0.1), emphasizing the efficient use of iron provided as colloids. Importantly, these differences in *S. rosetta* growth were unlikely due to indirect effects from the prey bacteria, as *E. pacifica* achieved the same cell density when provided either form of iron (Fig. 2C; Fig. S3E). The dissolution of iron from ferric colloids (3.43 ± 1.01 µM Fe) likely aided the growth of *E. pacifica* in those conditions (Fig. S3F). Notably, thecates also grew better than slow swimmers when supplemented with ferric chelates that have lower reduction potentials than ferric EDTA (63, 64), such as ferric EGTA (Fe^3+^-ethyleneglycoltetraacetic acid) and ferric pyoverdine (Fig. S3D), indicating that thecates may better acquire ferric cations tightly bound to bacterial siderophores. Overall, these experiments demonstrated cell-type-specific differences between thecates and slow swimmers in assimilating ecologically relevant sources of iron.

To derive iron from ferric colloids for enhanced cell growth, thecates can directly ingest iron particles. We visualized ferric colloids by embedding them with a fluorescently labeled polysaccharide. In time-lapse images, thecates captured ferric colloids on their collar before phagocytosing the particles (Fig. 2D; Fig. S4; Movie S1). Confocal sections showed that ferric colloids localized inside of the cell to acidic vacuoles that were marked with a pH-sensitive dye (Fig. 2E; Fig. S4D). The fluorescence in those vacuoles was primarily due to the accumulation of ferric colloids and not autofluorescence, as thecates provided with fluorescent iron colloids exhibited more intense fluorescence than cells that were not provided any iron colloids (Fig. 2F, *t*-test, *P* = 0.03). Thus, phagocytosis enables *S. rosetta* to directly acquire and accumulate insoluble iron.

We next tested if the cell-type-specific expression of *cytb561a* was necessary for the increased proliferation of thecates in the presence of ferric colloids. With an improved genome-editing pipeline (Fig. S5; see Materialsl and Methods), we altered *cytb561a* expression by introducing a premature termination sequence (65) at position 151 of *cytb561a* to incorporate nonsense mutations and a polyadenylation signal (Fig. 3A; Fig. S6), which diminished *cytb561a* mRNA in thecates by 3.6-fold compared with wild-type (Fig. S7D, Tukey’s multiple comparison, *P* = 0.004). This mutant allele, *cytb561a^PTS^*, eliminated the difference in cell proliferation between slow swimmers and thecates grown with 100 µM ferric colloids (Fig. 3B, two-way ANOVA, *P* > 0.1). When *cytb561a^PTS^* was mutated to restore protein expression with a synonymous allele (*cytb561a^G152A^*), thecates with wild-type and *cytb561a^G152A^* alleles displayed the same growth advantage in the presence of ferric colloids (Fig. S7E, Tukey’s multiple comparison, *P* = 0.2533). A comparison of *cytb561a^PTS^* and wild-type growth dynamics (Fig. S8) showed that the enhancement of thecate growth with ferric colloids was primarily due to a 2-fold larger carrying capacity and a shorter doubling time (17.9 h vs 13.2 h, two-way ANOVA, *P* = 0.035).

Although *cytb561a* was necessary for the enhancement of thecate growth in the presence of iron colloids, the induction of *cytb561a* expression in the thecate transcriptomes may also have been due to differences between the PRA-390 and HD1 strains that were respectively used to generate slow swimmer and thecate transcriptomes or due to differences between the nutrient content of the media used to culture slow swimmers versus thecates. To distinguish among these possibilities, we measured *cytb561a* mRNA expression in slow swimmers and thecates derived from a common strain (PRA-390) and cultured with either 100 µM ferric colloids or ferric EDTA. With either source of iron, the expression of *cytb561a* mRNA increased in thecates compared with slow swimmers (Fig. 3C; Fig. S7): 89-fold with ferric EDTA and 66.2-fold with ferric colloids (two-way ANOVA, *P* ≤ 0.0014). To further examine the regulation of *cytb561a* expression, we engineered a strain of *S. rosetta* at the endogenous locus of *cytb561a* to introduce a carboxy-terminal epitope tag (ALFA-tag [66] for measuring protein levels; Fig. 3A; Fig. S6). From this strain, we differentiated slow swimmers and thecates and then grew these strains in iron-replete or iron-deficient media (Fig. 3D; Fig. S9; Table S2). Cytb561a was undetectable in slow swimmers, mirroring the low expression of *cytb561a* transcripts. In thecates, Cytb561a levels were elevated in iron-depleted conditions but were so variable that we could not conclude (*t*-test, *P* > 0.1) if *S. rosetta* may have post-transcriptional mechanisms to regulate iron homeostasis as seen in animals and other eukaryotes (67). Nonetheless, the levels of Cytb561a and *cytb561a* transcripts both support the conclusion that thecates are a stable cell type that expresses *cytb561a* as part of its differentiation regulon. In combination with growth assays, these results suggest that the increased expression of *cytb561a* in thecates confers a cell-type-specific function to assimilate iron from insoluble ferric colloids for faster growth to higher cell densities.

### Cytb561 paralogs possess distinct biochemical properties

Although the expression of *cytb561a* in thecates was necessary for improved growth in the presence of colloidal iron, slow swimmers and thecates with the *cytb561a^PTS^* mutation could still grow—even if that growth was impaired (Fig. 3B). These observations and the relatively steady mRNA expression of *cytb561b* and *cytb561c* across cell types (Fig. 1C) would be consistent with these paralogs sustaining a baseline ability to acquire iron. However, these paralogs may not only be functionally redundant but also have evolved differences in their iron reduction activity.

**Fig 4:**
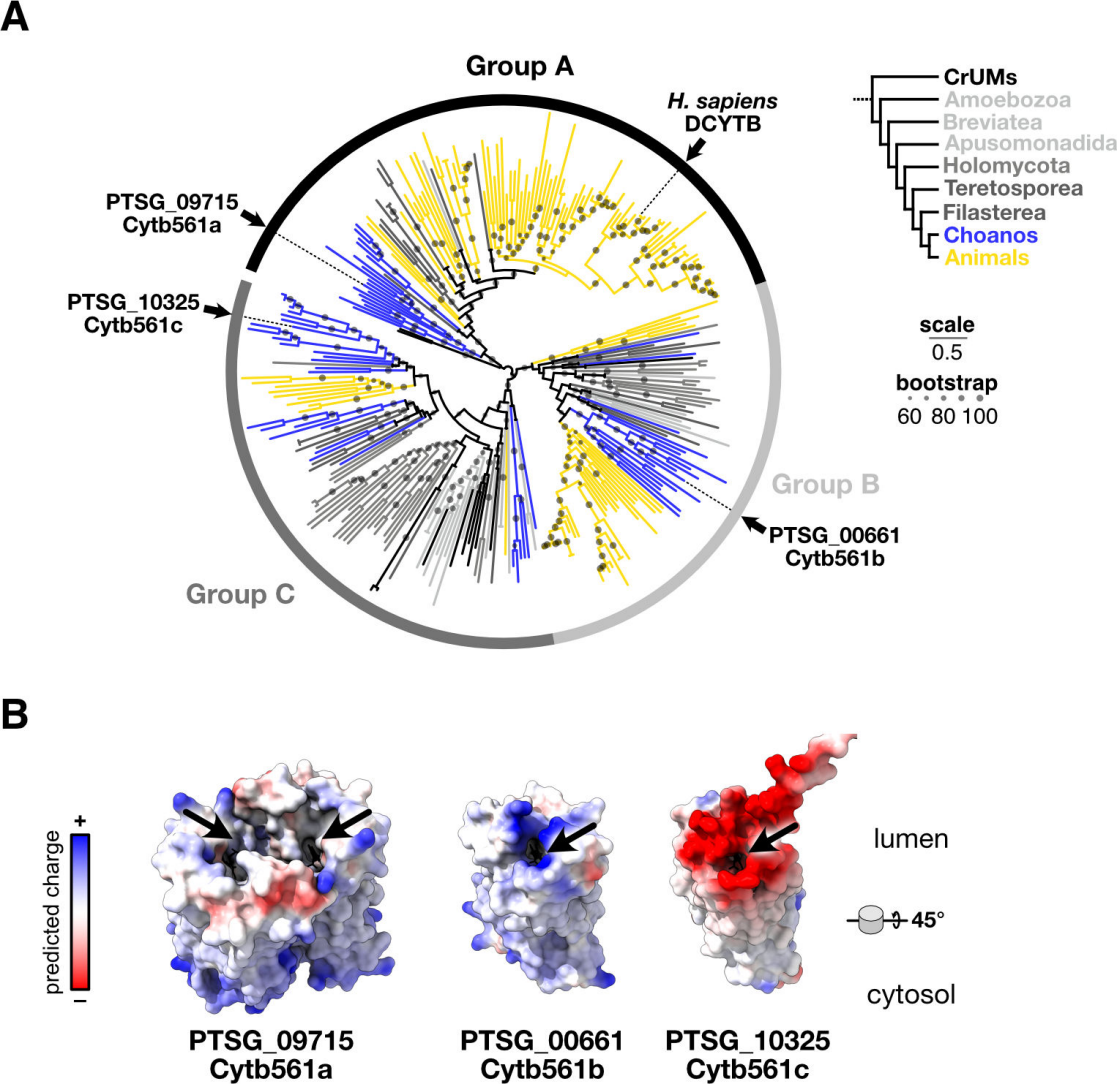
Cytb561 paralogs possess distinct biochemical properties. (**A**) Phylogeny of iron reductases reveals that Cytb561a is an ortholog of animal DCYTB. Each paralog of *S. rosetta* falls into distinct clades of cytochrome b561. Circles on each branch are proportional to bootstrap values greater than sixty. Scale bar indicates the average number of substitutions per site. Branch colors correspond to clades as shown in the legend. (**B**) Predicted structures of *S. rosetta* Cytb561 paralogs show differences in dimerization and substrate-binding interfaces. Alphafold (version 3) predictions show Cytb561a as the only paralog-forming homodimers. Predicted electrostatic surfaces Cytb561 paralogs show different charge distributions around the substrate binding pocket (arrows). Models are angled 45° to view the lumenal surface where iron binds.

To better compare the cytochrome b561 paralogs in *S. rosetta*, we first built a phylogenetic tree (68) from an alignment (69) of single-domain Cytb561 proteins found in Amorphea (Fig. 4A), a eukaryotic supergroup that includes Holozoa and Amoebae (70, 71). The unrooted tree of cytochrome b561 proteins robustly supported (ultrafast bootstrap [72] > 88) three distinct groups. Groups B and C contained protein sequences from each major clade in Amorphea and its sister group CRuMs (Collodictyonida, Rigifilida, and Mantamonadida). Strikingly, sequences from Holozoa dominated Group A, as a single protein from Amoebozoa was the only sequence from outside of Holozoa that fell into this group. Notably, Group A not only contained mammalian DCYTB proteins but also Cytb561a from *S. rosetta*, supporting the annotation of *cytb561a* as a *DCYTB* ortholog. The Cytb561b and Cytb561c proteins from *S. rosetta* fell into Groups B and C, respectively.

Because each *S. rosetta* paralog was part of a different group of cytochrome b561 proteins, we examined (73) multiple sequence alignments and their predicted structures (74) (Fig. S10 to S13) in the context of the human DCYTB crystal structure (75) to evaluate if the deep evolutionary divergence between paralogs was reflected in their biochemical characteristics (Fig. 4B). DCYTB forms a homodimeric complex with each monomer folding into six transmembrane helices that sandwich two heme groups in the interior of the protein. These heme groups mediate the transfer of an electron from an ascorbate molecule on the cytosolic side of the protein to ferric cations on the lumenal side. Thus, in our comparisons of multiple sequence alignments, we focused on the residues in DCYTB that bind ascorbate, mediate homodimerization, and attract iron. First, all homologs appear to have conserved positively charged residues that form the pocket to bind negatively charged ascorbate (75) (Fig. S10). Second, only proteins from Group A conserve residues that mediate dimerization (Fig. S11), which was also reflected in predicted dimeric structures of paralogs from *S. rosetta* (Fig. S12). Third, among the cytochrome b561 groups, we noticed significant differences on the lumenal surface that forms the binding site for ferric cations (Fig. 4B, arrow and S13). Homologs within Group A, which contains Cytb561a and DCYTB, possess a mix of positive and negative charges, indicating Group A paralogs can bind iron and coordinated ligands (75). Group C homologs have largely anionic surface charges at the putative iron binding site, which could plausibly bind iron, as seen by the ferric reductase activity of mouse homologs belonging to this group (76). However, the positively charged binding pockets conserved across Group B homologs may repel rather than bind iron cations. For example, Group B paralogs from humans and mice reduce monodehydroascorbate as an ascorbate regeneration mechanism (77). Notably, the differences in charge distribution among cytochrome b561 groups appear to be general features of those groups (Fig. S13C); however, electrostatic properties can diverge within those lineages (Fig. S13D). With each paralog from *S. rosetta* possessing distinct biochemical properties near the iron-binding site, the improvement in thecate growth with ferric colloids may not only be due to the mass action from more Cytb561 proteins but also through the distinct biochemical activity of Cytb561a.

### Phylogenetic and geographic distribution of Cytb561 homologs from choanoflagellates

Using the insights gained from studying the role of Cytb561a for assimilating iron in *S. rosetta*, we examined the conservation of cytochrome b561 homologs in choanoflagellates and their distribution in global oceans to infer how diverse choanoflagellates may acquire iron. First, we associated the presence of Cytb561 homologs with morphological and environmental traits. Second, we used metagenomic surveys of the open ocean to map the location of Cytb561 homologs from choanoflagellates. Together, these analyses illustrate that Cytb561 homologs from choanoflagellates are widespread throughout the global oceans with particular clades driving the abundance of certain homologs due to lineage-specific gains and losses.

**Fig 5:**
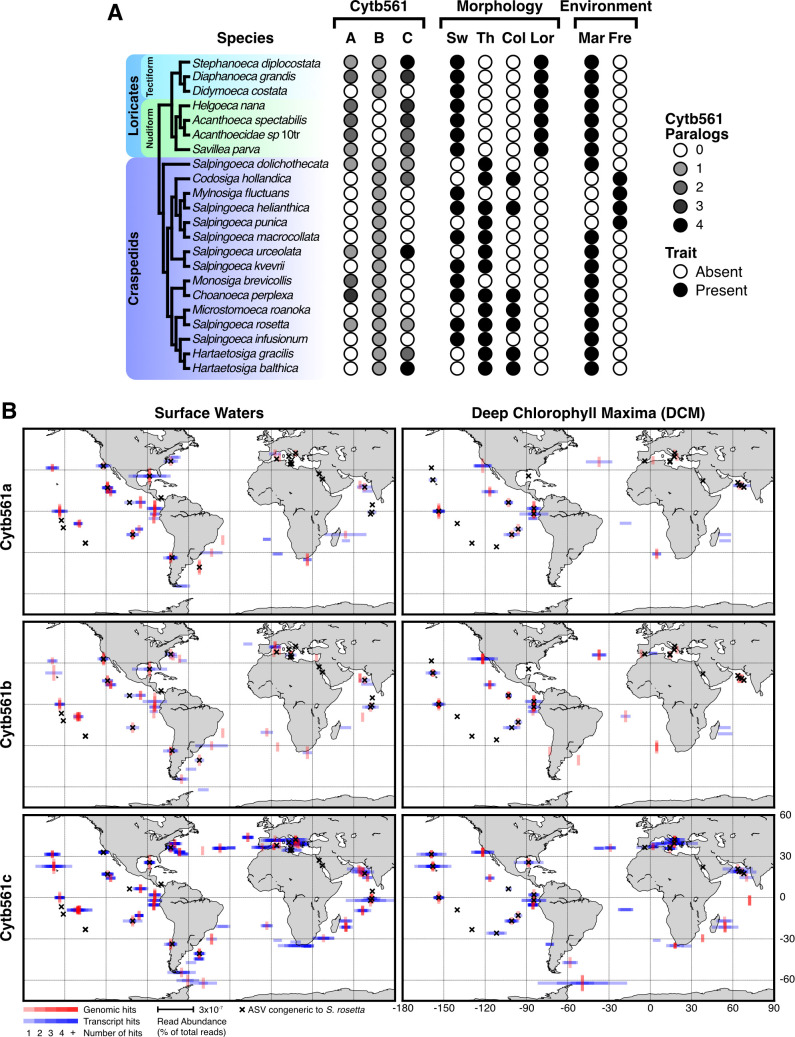
Phylogenetic and geographic distribution of Cytb561 homologs from choanoflagellates. (**A**) The presence of Cytb561 homologs across choanoflagellate species. Choanoflagellates are divided into two major groups: Loricates, including Tectiform and Nudiform species, are choanoflagellates that produce siliceous coverings, and Craspedids, in which *S. rosetta* belongs, have organic coverings. The grayscale corresponds to the number of homologs found in the phylogenetic tree in Fig. 4A. Morphology is determined by the observation of cell types in culture (46, 83): Sw for swimming cells, Th for theca formation, Col for colony formation, Lor for lorica or siliceous covering formation. Environment corresponds to the original isolation location and culturable media salinity (Mar for marine and Fre for freshwater) (46, 83). Tree order and species characteristics adapted from (83) and (46). (**B**) The geographic distribution of Cytb561 homologs. Homologs and congeners of *S. rosetta* were identified from a repository of metagenomes and metatranscriptomes from marine environments. In these data sets, surface waters are defined as the top 5 m of the ocean, and Deep Chlorophyll maxima (DCM) are found 20–200 m from the surface, depending on the highest intensity from chlorophyll. Homolog abundance was measured as the percentage of total reads from each sample site. Red vertical lines correspond to genomic abundance. Blue horizontal lines correspond to transcript abundance. Black crosses denoted sites where ASVs congeneric to *S. rosetta* were detected. Overlapping hits at matching locations increase the color saturation. Longitude and latitude degrees are marked on the bottom right map.

We first compared the number of Cytb561 homologs (Fig. 4A) to morphological and environmental features of twenty choanoflagellate species transcriptomes (46) and two that also have genomes (44, 78) (Fig. 5A). These choanoflagellates cover the major choanoflagellate taxa: Loricates, including Tectiform and Nudiform species, are choanoflagellates that produce siliceous coverings, and Craspedids, in which *S. rosetta* belongs, have organic coverings. When we examined the homolog distribution among those lineages, the number of Cytb561a and Cytb561c paralogs expanded in Loricates compared with Craspedids. Nudiform Loricates also lacked Cytb561b. Furthermore, all freshwater choanoflagellates, which are all Craspedids, lacked Cytb561a. The absence of homologs in certain lineages may be due to the transcriptomes failing to capture all cell-type-specific gene expression; however, taken at face value, the variations in the number of homologs may indicate lineage-specific divergences in iron acquisition processes due to environmental factors or secondary metabolic processes. For example, freshwater environments are replete with iron (79), which could relieve the pressure to harvest iron. Furthermore, silicon utilization in loricates derived from a more recent horizontal gene transfer (80), and the biochemical consequences may have altered iron acquisition mechanisms because iron limitation can decrease the biological uptake of silicon in diatoms (81, 82).

Because iron availability tunes the productivity of marine ecosystems (84), we examined the presence and expression of Cytb561 homologs in the repository Ocean Gene Atlas (85) of metagenomes and metatranscriptomes collected by the Tara Ocean expedition (86). When we visualized the distribution of choanoflagellate Cytb561 homologs (Fig. 5B), we found that they varied in location, depth, and abundance (defined as the percentage of reads corresponding to a given gene from total genomic [red] or transcriptomic [blue] reads at each site). In these data, homologs were more often found in samples from the surface and the deep chlorophyll maxima (DCM, depth where chlorophyll concentrations are highest, 20–200 m [87]). Each homolog was more abundant in surface waters compared with the DCM, indicating that choanoflagellates with these homologs are more prevalent in surface waters. The most abundant and cosmopolitan homolog was Cytb561c, whereas Cytb561a and Cytb561b were less abundant at both depths.

To ascertain if *S. rosetta* contributed to the expression of Cytb561 homologs in these data, we identified Cytb561 homologs that were most closely related to *S. rosetta* and searched a database (metaPR2) (88) for amplified sequence variants (ASVs) corresponding to the V9 variable region of the 18S rRNA of *S. rosetta*. Neither the Cytb561 homologs nor the ASVs matched *S. rosetta*; hence, we infer these sequences come from species congeneric to *S. rosetta* rather than *S. rosetta* itself (Fig. S14A and B). These species were widely dispersed in the open ocean (Fig. 5B; Fig. S14C), and Cytb561a was the most abundant in samples from the surface and DCM. The sites where Cytb561a is expressed may indicate the presence of thecates if congeners of *S. rosetta* have similar cell-type expression programs.

The elevated abundance of congeneric Cytb561a homologs in the open ocean lead us to examine the prevalence of iron at the surveyed locations using a biogeochemical model (PISCES, v2) (61, 89) of dissolved (< 0.2 µm) and small particulate (0.2–35 μm) (90) iron species. Both classes of iron are present in locations where Cytb561a homologs are detected, and seasonal variations in iron may impact the abundance of Cytb561a at those locations (Movies S2 and S3). Taken together with our experimental observations (Fig. 2 and 3), the overlap between Cytb561a expression and environmental sources of iron indicates locales where choanoflagellates may better assimilate iron for their growth; however, further ecological studies would be necessary to fully integrate environmental and laboratory observations.

In the open ocean, dissolved and particulate iron levels display large temporal and geographical fluctuations, but the concentration range in the open ocean (61) (pM-nM) is much lower than in intertidal zones (91) and estuaries (92) (µM–mM). *S. rosetta* was isolated once from tidal mud flats off the coast of Virginia, USA (37.45278° N 75.67521° W), as a colonial rosette (21). In such tidal ecosystems, relatively iron-rich freshwaters deposit iron in a particulate form (93, 94) caused by iron precipitation when freshwater mixes with saline marine water (92, 95). The abundance of particulate iron in tidal and estuarial environments may have contributed to *S. rosetta*’s ability to utilize colloidal iron; however, the isolation of a rosette indicates *cytb561a* expression was not active. Thecates form and persist under nutrient limitation, and the eutrophic local environment likely favored other cell types, such as rosettes. Instead, cell differentiation of *S. rosetta* may allow for ecological plasticity across nutrient gradients outside of the environment from which it was isolated.

## DISCUSSION

Iron is an essential micronutrient affecting the growth and productivity of phytoplankton (84). Field measurements and ocean scale models have found that iron particulates are the major source of iron for 40% of the world’s surface ocean waters (61). In addition to photolytic reduction (8, 96) and bacterial siderophores (97, 98), the ingestion of iron particles by phagotrophic protists is a major mechanism to solubilize iron and can alleviate iron limitation for cell-walled organisms that rely on obtaining iron in soluble forms (4, 8, 99). As efficient phagotrophs (1, 3, 7, 12), choanoflagellates ingest iron particulates (Fig. 2D; Fig. S4); however, iron assimilation from ferric colloids depends on the expression of a specific Cytb561 paralog in only one cell type of *S. rosetta*. This dependency emphasizes the importance of integrating protein evolution (100) and gene expression during life history transitions (18, 35) to determine (101) the ecological roles of microeukaryotes in the acquisition and distribution of limiting nutrients throughout their surrounding environment.

The cell-type-specific expression of *cytb561a* not only illustrates how cellular differentiation impacts the environmental function of a choanoflagellate but also informs how cellular differentiation evolved as a defining feature of animal multicellularity (36, 37, 39–41, 102). Choanoflagellate cell types have been primarily defined by their morphology and motility (12, 21). In addition to those cell biological characteristics, we show here that thecates have a distinct ability to utilize ferric colloids through the induced expression of the iron reductase *cytb561a* (103–105). The coupling of this function to cell differentiation stands in contrast to the regulation of iron utilization in other microeukaryotes that transiently transcribe iron utilization genes in response to external iron concentrations (103–105)

The functional difference between slow swimmers and thecates more closely resembles the stable differentiation of cell types during animal development (106–108). Our observations support models for the origin of animal cell differentiation in which the last common holozoan ancestor differentiated into unique cell types at each life history stage (36, 37, 40, 42, 109). Evolution along the animal stem lineage coupled cell differentiation to multicellular development, resulting in multicellular bodies composed of functionally distinct cell types (110). Importantly, mechanisms to distribute resources from cells that acquire nutrients from the external environment (e.g., epithelial cells) (111) to the cells that reside internally helped to transform the functions of various cell types into a common organismal physiology (38).

## MATERIALS AND METHODS

### Culturing *S. rosetta*

To grow cell types of *S. rosetta* for the generation of transcriptomes, slow swimmer and rosettes were grown in 5% sea water complete (SWC) media (22, 112) (Table S2), seeded at 10^4^ cells/mL, and grown for 48 h at 22°C. Rosettes were induced with outer membrane vesicles (OMVs) harvested from the bacterium *Algoriphagus machipongonensis* (25). Fast swimmers were prepared the same way as slow swimmers but were grown for 72 h at 22°C and transitioned to 30°C for 2 h and 45 min. The aforementioned cell types were derived from strain PRA-390, whereas thecates were grown from the strain HD1, a strain which is constitutively in the thecate cell type (Table S4). Thecates were grown in 10% Cereal Grass Media 3 (CGM3) (22, 112) (Table S2), seeded at 10^4^ cells/mL and grown for 48 h at 22°C.

For iron acquisition experiments and standard culturing of *S. rosetta*, all cell types were derived from the strain PRA-390 and grown in 25% Red Algae (RA) media (27) (Table S2) with the feeder bacteria *E. pacifica*. Cultures were grown at 18°C for maintaining cultures and shifted to 27°C 1 day prior to conducting iron acquisition experiments.

### RNA extraction

In a previous paper (112), we developed a lysis buffer to extract RNA from *S. rosetta* that has subsequently been used in other work for RNA (29, 113, 114) and protein (115) extraction. Here, we show that this buffer was developed to preferentially lyse *S. rosetta* in cultures that have an abundance of the prey bacterium *E. pacifica*. To preferentially lyse *S. rosetta*, we reasoned that sterol-based detergents, such as digitonin, would more effectively disrupt membranes with sterols, like those of *S. rosetta* and other eukaryotes (116) rather than bacterial membranes that largely lack the sterols (117). Additionally, the lysis buffer contains RNase inhibitors (RNaseIN and heparin) and translation inhibitors to decrease the degradation of mRNA. We evaluated the efficacy of this buffer by tracking ribosomal RNA from *S. rosetta* and *E. pacifica* in lysed samples that were centrifuged to separate the supernatant and pellet, in which we found that *S. rosetta* ribosomal RNAs were enriched in the supernatant and bacterial ribosomal RNAs were enriched in the pellet (Fig. S1). The optimization of this procedure resulted in the following method to obtain RNA samples from each cell type of *S. rosetta*.

After counting the cell concentration in a culture of *S. rosetta* feeding on *E. pacifica*, a volume yielding 10^7^ cells was centrifuged at 2600 × *g* for 5 min at 4°C. The pellet from this sample was resuspended in 100 µL of preferential lysis buffer (20 mM Tris-HCl, pH 8.0; 150 mM KCl; 5 mM MgCl_2_; 250 mM sucrose; 1 mM DTT; 10 mM digitonin; 1 mg/mL sodium heparin; 1 mM Pefabloc SC; 100 µg/mL cycloheximide; one tablet/5 mL buffer EDTA-free Protease Inhibitor Tablet [Sigma Aldrich Cat. No. 11836170001]; 0.5 U/µL Turbo DNase [Thermo Fisher Scientific, Cat. No. AM2239]; and 1 U/µL SUPERaseIN [Thermo Fisher Scientific, Cat. No. AM2696]) and incubated on ice for 10 min. Cells were triturated through a 30G needle 10 times. Afterward, the insoluble debris was pelleted by centrifugation at 6,000 × *g* for 10 min at 4°C. The supernatant was removed and adjusted to a volume of 100 µL with RNase-free water. Total RNA was purified on a silica membrane with the RNA clean-up protocol from the RNAeasy kit (Qiagen, Cat. No. 74104). The protocol link is https://doi.org/10.17504/protocols.io.261ge4bojv47/v1.

### RNA library preparation and sequencing

In preparation for sequencing, the quality of total RNA from *S. rosetta* samples was assessed by Agilent Bioanalyzer 2100 (Fig. S1C) with the Agilent RNA 6000 Nano Kit (Agilent, Cat. No. 5067-1511). Then, total RNA (500 ng per sample) was purified by one round of polyA mRNA selection with oligo-dT magnetic beads (NEB, Cat. No. S1550S), converted to cDNA using KAPA mRNA HyperPrep kit (KAPA biosystems, Cat. No. KK8580) and indexed-adapter ligated with KAPA single-indexed adapter kit (KAPA biosystems, Cat. No. KK8701). Final library quality was determined by Bioanalyzer 2100 using the Agilent High Sensitivity DNA Kit (Agilent, Cat. No. 5067-4626) and pooled together after normalizing samples based on their quantity from qPCR. Sequencing was carried out by the QB3-Berkeley Genomics core (QB3 Genomics, UC Berkeley, Berkeley, CA, RRID:SCR_022170), on the Illumina HiSeq 4000. All samples were pooled and run on a single lane with 12.4 to 61.3 million reads per sample. Reads were demultiplexed and checked for quality by fastqc (Babraham Bioinformatics). Metadata for sequencing can be found in Table S1.

### RNA-seq quantification and gene enrichment analysis

RNA-seq reads from each sample were mapped to a reference *S. rosetta* transcriptome (https://ftp.ensemblgenomes.ebi.ac.uk/pub/protists/release-58/fasta/protists_choanoflagellida1_collection/salpingoeca_rosetta_gca_000188695/cdna/) using kallisto (118). Transcript abundance and differential expression were quantified with sleuth (119). Transcript abundance measurement can be found at https://doi.org/10.6084/m9.figshare.28225106. Gene enrichment for thecates was quantified by selecting genes with *q* < 0.01 and log_2_ (fold change + 0.5) >1. The resulting genes were assigned GO terms using DAVID Bioinformatics (50, 51) where *S. rosetta* gene IDs could be identified by “ENSEMBLE_GENE_ID”. For ease of interpretation, assigned GO terms were collapsed down using REVIGO (120) and further assigned to functional categories in *S. rosetta* (TaxID: 946362) by GO Slim (52) (https://doi.org/10.6084/m9.figshare.28225106).

### *S. rosetta* growth with different sources of iron

#### Media preparation

To test the ability of *S. rosetta* cell types to differentially utilize iron sources, we developed a method to culture *S. rosetta* under iron limitation. We created an iron-depleted media formulation named 4% peptone glycerol (PG) medium (Table S2). We added iron to this medium from two different sources: ferric EDTA was prepared by dissolving FeCl_3_ and EDTA to a final concentration of 1.46 mM in artificial seawater (ASW [Ricca Chemical Company, Cat. No. R8363000-20F]) and sterile filtering through a 0.22 µm polyethersulfone (PES) filter. Ferric colloids were prepared by adding a small volume of 70% (vol/vol) ethanol to FeCl_3_(*s*) to sterilize the solid; after evaporating the liquid in a biological safety cabinet, 1.46 mM FeCl_3_ was prepared in ASW. The solution was heated to 50°C for 10 min to precipitate ferric (oxyhydr)oxides or ferric colloids (60).

#### Fluorescent ferric colloid preparation

Ferric colloids were prepared as above, except during the 50°C incubation, a fluorescent dextran (10 kDa anionic dextran conjugated to tetramethylrhodamine [Thermo Fisher, Cat. No. D1868] for time-lapse microscopy, and 10 kDa anionic dextran conjugated to Alexa Fluor 647 [Thermo Fisher, Cat. No. D22914] for confocal microscopy) was added to a final concentration of 20 µg/mL. This dextran co-precipitated with the ferric colloids, thereby embedding fluorophores within the colloids. The precipitated ferric colloids were pelleted by centrifugation at 4,600 × *g* for 10 min at room temperature. The pellet was resuspended in ASW supplemented with 2% (vol/vol) dextranase (Sigma Aldrich, Cat. No. D0443) to digest any excess dextran during a 10 min incubation at 60°C. After pelleting the ferric colloids again, they were resuspended to ~1.46 mM in ASW. Finally, the resuspended colloids were ultrasonicated for two, 1 min cycles (pulsed for 1 s on, 1 s off) to create smaller particles for easier feeding and imaging, as larger particles broke down slowly and often obscured cells during imaging.

#### Determining iron concentration in media

To determine the background concentration of soluble iron in our newly formulated media and the amount of labile iron liberated from the ferric colloids, we used a modified protocol (4) to measure soluble iron concentrations in 4% PG and 4% PG with 100 µM ferric colloids. To measure these iron concentrations, 50 mL of 4% PG media was incubated at 27°C for 6 h with and without 100 µM ferric colloids. The medium was then centrifuged at 4,600 × *g* for 15 min at room temperature. The top 45 mL of the supernatant was taken for the following steps. To capture and concentrate the soluble iron in the supernatant, 125 µM 8-hydroxyquinoline (oxine) was added to chelate labile iron, and 5 mL of 1 M MES, pH 6, was added to aid in oxine’s solubility in the media and its binding to iron, which is facilitated by lower pH. These solutions were incubated for 24 h protected from light and then filtered through a Sep-Pak C18 column (Waters, Cat. No. WAT036800) to bind oxine-iron complexes. The column was then washed with 5 mL water and eluted with 1.4 mL methanol. To measure iron concentrations in the eluate, 50 µL of the eluate was measured with a colorimetric iron detection kit (Sigma Aldrich, Cat. No. MAK025-1KT) following the provided protocol. Iron concentration was determined by measuring absorbance at 595 nm on a plate reader.

#### Iron-limited *E. pacifica* pellets

Iron-limited *E. pacifica* pellets were prepared by growing *E. pacifica* in 4% PG for 72 h at 225 rpm and 30°C, then pelleted, aliquoted, and frozen into 10 mg pellets, and stored at −80°C. *E. pacifica* pellets were resuspended in 1 mL of media to achieve a 10 mg/mL liquid bacteria stock to add to cultures.

#### Culturing with different iron sources

To set up the iron utilization assay, cultures grown in 25% RA were passaged at a 1:60 dilution into 25% RA and grown for 72 h at 18°C. This culture was then passaged at a 1:60 dilution into 25% RA and grown for 24 h at 27°C. Finally, this culture was passaged at a 1:30 dilution into 4% PG (which had no iron added) and grown at 27°C for 24 h. Cells from this final passage were twice washed by first centrifuging the culture at 2,600 × *g* for 4 min and then resuspending the pellet in 30 mL sterile ASW. After the final wash, the pellet was resuspended in 100 µL ASW and counted. These washed cells were used to seed cultures at 10^4^ cells/mL in 4% PG with the additions of 100 µM iron (ferric EDTA or ferric colloids) and 50 µg/mL of iron-limited *E. pacifica* pellets (see above). The resulting culture was grown at 27°C for 48 h and harvested for each experiment.

### qPCR

#### Primer design

Primers were designed against *cytb561a,* the experimental gene of interest, and *cofilin,* a highly expressed eukaryotic-specific housekeeping gene with a consistent expression between *S. rosetta* cell types, as shown by the RNAseq analysis. Primers for quantitative polymerase chain reaction (qPCR) were designed by selecting 19–20 bp oligos spanning exon-exon junctions from cDNA sequences (downloaded from https://protists.ensembl.org/Salpingoeca_rosetta_gca_000188695/Info/Index). In the case of *cytb561a,* primers were selected downstream of the premature termination sequence edited by CRISPR/Cas9.

#### Primer validation

Target amplicons were amplified by polymerase chain reaction (PCR) from the cDNA of the slow swimmer and thecate cultures (Fig. S6) with the Luna Universal qPCR Master Mix (NEB, Cat, No. M3003). The amplicons were run on a 1% (wt/vol) agarose gel in Tris-Borate-EDTA (TBE) buffer to verify that the primer sets amplified only one amplicon. The amplification efficiency of primer sets was characterized across a serial dilution of ssDNA standards for each target (Fig. S6). ssDNA standards were generated from PCR products amplified with a forward primer that was 5’ phosphorylated to promote Lambda exonuclease digestion of that strand (NEB, Cat. No. M0262S) and a reverse primer with phosphorothioate bonds between the first four 5’ nucleotides to block digestion. Those PCR products were then digested with Lambda exonuclease following NEB’s protocol. ssDNA concentrations were determined by Qubit ssDNA Assay Kit (Thermo Fisher, Cat. No. Q10212) by creating a standard curve with the provided standards and then reading fluorescence (Ex = 500 nm/Em = 540 nm) on a plate reader (Molecular Devices, SpectraMax iD5). The ssDNA standards were serially diluted from 10 (20) to 10 copies/µL in a solution with 10 ng/µL of *S. rosetta* RNA (Total RNA after removing mRNAs) to account for any matrix effects.

#### cDNA preparation

Samples from slow swimmer or thecate cultures were pelleted and lysed using the preferential lysis protocol, and total RNA was purified by RNeasy MinElute Cleanup Kit (Qiagen, Cat. No. 74204). cDNA was prepared by SuperScript IV Reverse Transcriptase (Thermo FIsher, Cat. No. 18090010) following the provided protocol using oligo d(T)_20_ for synthesis. However, the incubation temperature for cDNA synthesis was increased to 60°C to account for the high GC content of the *S. rosetta* genome. The protocol link is https://dx.doi.org/10.17504/protocols.io.ewov1nnzogr2/v1.

#### qPCR

Three microliters of cDNA samples or ssDNA standards was added to a 20 µL reaction with Luna Universal qPCR Master Mix (NEB, Cat, No. M3003) according to the provided protocol. The samples were amplified in a single run on the QuantStudio 3 Real-Time PCR System (Thermo Fisher, Cat. No. A28567). The protocol link is https://dx.doi.org/10.17504/protocols.io.n2bvjnjongk5/v1.

### Genome editing

#### Culturing cells for transfection

Mutant strains were generated with a modified protocol from a previously published method (65). Cultures of *S. rosetta* slow swimmers were maintained in 15% RA/2% PYG (called 15/2, Table S2) at 22°C, and 48 h prior to transfection, the cultures were seeded at 10^4^ cells/mL in 80 mL of 15/2 media in 300 cm^2^ vented tissue culture flasks (VWR, Cat. No. 10062-884) and grown at 22°C.

#### Cas9 RNP preparation

On the day of transfection, Cas9 bound to a guide RNA (Cas9 RNP) was prepared by combining 2 µL of 100 µM guide RNA (gRNA) with 2 µL of 20 µM EnGen SpyCas9 NLS (NEB, Cat. No. M0646T) and incubated at room temperature for 2–4 h. (Note: our gRNA was ordered as synthetic RNA oligonucleotides from Integrated DNA Technologies that came as an Alt-R CRISPR-Cas9 crRNA and an Alt-R CRISPR-Cas9 tracrRNA. These two oligonucleotides were annealed together to form gRNA.) Guide sequences can be found in Table S3.

#### Transfection

During the Cas9 RNP incubation, the cells were harvested for transfection by centrifuging the cultured cells at 2,400 × *g* for 3 min at 4°C. The supernatant was discarded, and the cells were resuspended in 50 mL of cell wash buffer (420 mM NaCl; 50 mM MgCl_2_; 30 mM Na_2_SO_4_; 10 mM KCl; titrated to pH 8.0 with ~2.4 mM NaHCO_3_). The cells were centrifuged again at 2,400 × *g* and 4°C for 3 min. The supernatant was removed, and the pellet was resuspended in 100 µL of cell wash buffer. Cells were counted and diluted to 5 × 10^7^ cells/mL, split into 100 µL aliquots, and centrifuged for 2 min at 800 × *g* and room temperature. The supernatant was removed and replaced with 100 µL priming buffer (40 mM HEPES-KOH, pH 7.5; 34 mM lithium citrate; 15% (wt/vol) PEG 8000; 50 mM cysteine; 1.5 µM papain). Cells were primed for 45 min at room temperature. Afterward, the priming was quenched with 10 µL of 50 mg/mL bovine serum albumin. The primed cells were centrifuged at 1,200 × *g* for 4 min at room temperature. After removing the supernatant, the cells were resuspended in 25 µL ice-cold SF buffer (Lonza Cat. No. V4SC-2096). Nucleofection reactions were prepared by combining 16 µL ice-cold SF buffer, 4 µL Cas9 RNP, 2 µL of repair oligo, and 2 µL of the resuspended and primed cells. This reaction mix was loaded into a 96-well nucleofection plate (Lonza Cat. No. AAF-1003S, AAF-1003B) and pulsed with CU 154 (Fig. S4). Hundred microliters of ice-cold recovery buffer (10 mM HEPES-KOH, pH 7.5; 900 mM sorbitol; and 8% [wt/vol] PEG 8000) was added immediately to each pulsed well and incubated for 5 min at room temperature. Afterward, the entire content from each nucleofection well was transferred into 2 mL of 15/2 media with the addition of 50 µg/mL *E. pacifica* pellet. The protocol link is https://dx.doi.org/10.17504/protocols.io.j8nlk86o5l5r/v1.

### Strain isolation and screening

We adapted the Cas12a DETECTR genotyping assay (121) to screen for cells with desired mutations from Cas9 genome editing. Following overnight recovery post-nucleofection, cells were counted and diluted to 45 cells/mL in 10% RA (Table S2) with 50 µg/mL *E. pacifica* pellet and aliquoted into 96-well plates. Plates were then grown at 27°C for 72 h to propagate cells. Afterward, 12 µL from each well was added into 36 µL DNAzol Direct (20 mM potassium hydroxide; 60% [wt/vol] PEG 200, pH 13.3–13.7 [note: it is important to test a range of pH values to establish the optimal pH for your own use]) that was pre-aliquoted into 96-well PCR plates, which we called the lysed sample. The lysed samples were then incubated at 80°C for 10 min. The target DNA was amplified (primer sequences in Table S3) in PCRs prepared with GoTaq Clear Master Mix (Promega, Cat. No. M7123) using 2 µL of the lysed samples per 25 µL PCR. Cycling conditions followed Promega guidelines and melt temperatures for oligos were calculated using https://www.promega.com/resources/tools/biomath/tm-calculator/. During the PCR, a Cas12a mastermix was assembled in two parts. First, Cas12a RNP (13 µL water; 2 µL r2.1 buffer [NEB, Cat. No. B6002S]; 2.5 µL 100 µM gRNA [ordered as a synthesized oligo]; 2 µL 100 µM LbaCas12a [NEB, Cat. No. M0653T]) was incubated for 5 min at room temperature. Second, the Cas12a RNP was combined with the rest of the components (for one 96-well plate: 486 µL water; 60 µL r2.1 buffer; 18 µL Cas12a RNP; 36 µL 5 µM ssDNA probe [IDT, Cat. No. 11-04-02-04]) and then incubated for 5 min at room temperature. Five microliters of the mastermix was added to each 25 µL PCR and incubated at 37°C for 1 h. The fluorescent signal was then measured in the VIC channel on a QuantStudio 3 Real-Time PCR System. Wells with high signal (≥10-fold above background) were recovered in 25% RA overnight at 27°C, counted, diluted to 3 cells/mL in 10% RA with 50 µg/mL *E. pacifica* pellet, and plated in a 96-well plate with 100 µL/well, which corresponds to 0.3 cells/well. Plates were grown at 27°C for 72–96 h. Finally, these clonal isolates were screened by digesting amplicons with a restriction enzyme (RE). Twelve microliters per isolate was added to 36 µL DNAzol, incubated at 80°C to make a lysed sample; 2 µL of the lysed samples was added to 25 µL PCRs with GoTaq Green mastermix (Promega, Cat. No. M7123). After amplification, 1 µL of RE was added to each 25 µL PCR and incubated following manufacturers’ protocols. RE digestions were run on 1% agarose gels with wild-type or undigested controls. For positive RE digestion hits, PCR products were Sanger sequenced to confirm the sequence of the edited site (Table S4). The protocol link is https://dx.doi.org/10.17504/protocols.io.14egn6n2pl5d/v1.

### Growth curves

We regularly monitored the cell density of *S. rosetta* over 48 h to assess the population dynamics of wild type and *cytb561^PTS^* thecates in iron utilization assay conditions. Cells were plated in 24-well plates with 100 µM ferric EDTA or ferric colloids sources in 4% PG or in nutrient replete media (25% RA) as a control. At each time point, the entire contents of one well were harvested by scraping the bottom and then transferring the liquid to a microfuge tube. Cells were fixed with 5 µL of 37.5% paraformaldehyde (PFA) and then counted on a Reichert Bright-Line Metallized Hemacytometer (Hausser Scientific, Cat. No. 1483). Growth curves were fit to a logistic growth equation that explicitly models the lag time with a Heaviside step function (27).

*E. pacifica* growth curves were set up in 4% PG media inoculated with 50 µg/mL iron-limited *E. pacifica* pellet and a ferric EDTA titration in clear bottom 96-well plates and grown for 48 h at 27°C. Growth was assessed by monitoring the optical density at 600 nm on a plate reader with orbital shaking before every reading. Ferric colloids were not included in the growth curves as iron particulates would shift optical density readings. To assess *E. pacifica* growth with ferric colloids, 4% PG media was inoculated with 50 µg/mL iron-limited *E. pacifica* pellet and 100 µM ferric EDTA or ferric colloids in 6-well plates and grown for 48 h at 27°C. Wells were scraped and 1 mL aliquots were centrifuged at 500 × *g* for 2 min to settle iron particulates. The top 500 µL of supernatant was gathered for measuring the optical density at 600 nm.

### Microscopy

#### Time-lapse of ferric colloid ingestion

Hundred microliters of 1.46 µM fluorescent ferric colloids were added to 2 mL of PRA-390 thecate cultures, previously grown in glass-bottomed dishes (World Precision Instruments, Cat. No. FD35-100) for 24 h at 22°C. Samples were imaged by widefield microscopy. The microscope was a Nikon Eclipse Ti2-E inverted microscope outfitted with a D-LEDI light source, Chroma 89401 Quad Filter Cube, and 60× CFI Plan Apo VC NA1.2 water immersion objective. Time-lapse images were acquired every 4 s over 2 h with 50 ms exposure time for brightfield and fluorescent (TRITC channel) images. In three independent experiments, a total of four feeding events were found in a field of 200 cells, although this was not an exhaustive search for ferric colloid intake frequency. One cell that was oriented on its side to best visualize feeding was selected for further processing. Images were processed in FIJI by cropping individual cells in 100 × 100 pixel bounds, and image contrast was manually adjusted. Ferric colloid particles were automatically tracked, first by setting a threshold to produce a binarized image (image >adjust > manual threshold) and then by finding particles (analyze > analyze particles > size = 10–300 pixels, circularity = 0.2–1.0).

#### Confocal microscopy of ferric colloid ingestion

One hundred microliters of 1.46 µM fluorescent ferric colloids was added to 2 mL of PRA-390 thecate cultures, previously grown in glass-bottomed dishes (World Precision Instruments, Cat. No. FD35-100) for 24 h at 22°C and incubated with the ferric colloids for 60 min prior to imaging. Immediately before imaging, cells were stained with 2 µM LysoTracker Red DND-99 (Thermo Fisher Scientific Cat. No. L7528) and 100 nM Memglow 488 (Cytoskeleton, Inc Cat. No. MG01-02). Samples were imaged by confocal microscopy on a Nikon Ti-E inverted microscope with a 60× Oil Plan Apo objective, Hamamatsu Quest camera, and a VT-iSIM super-resolution module. Images were acquired with a 200 ms exposure in the 488, 561, and 647 channels using µManager controller software (122). In three independent experiments, at least three fields of 30 cells each were captured across experimental and control conditions. Images were deconvolved using the Richardson-Lucy maximum likelihood algorithm implemented by Microvolution Fiji/ImageJ plugin (Microvolution, https://www.microvolution.com/products/imagej) with 10 iterations of 10% background correction. Representative images of cells were processed in Fiji by background subtracting with 200 pixel sliding paraboloids, cropping single cells in 150 × 200 pixel bounds, and manually adjusting image contrast and brightness. Ferric colloid internalization was measured in z-projected sum of slices of raw image stacks. Regions of interest (ROIs) were identified by thresholding the 561 nm channel, which detected lysosomes/food vacuoles, and then measuring mean fluorescence intensity in the 647 nm channel, which detected fluorescently labeled colloids. Raw fluorescent values of ROIs from three images from each independent replicate were collected and then fit to a gamma distribution to account for the non-gaussian fluorescence distribution. The mean of the gamma distribution of each replicate was then averaged for a global mean.

### Western blots

Cultures were grown in 25% RA or 4% PG (without supplemented iron) for 24 h at 27°C to test whether external iron conditions (replete or deplete) resulted in any post-translational regulation of protein expression. Cultures were scraped and then lysed according to the preferential lysis protocol, with the following modifications: TurboDNase and SUPERasein were replaced with 15 µL of Pierce universal nuclease (Thermo Scientific Cat. No. 88702) per 1 mL buffer, and heparin and cycloheximide were removed. After clearing the lysate, Tween-20 was added to a final concentration of 1% (vol/vol), and the sample was incubated for 10 min at room temperature to aid in liberating membrane-bound proteins. Protein concentration was determined by Bradford assay (Thermo Fisher Scientific Cat. No. 23236), and the samples were normalized to the lowest concentration. To prepare for SDS-PAGE, approximately 20 µg or 40 µg of total protein for thecates or slow swimmers, respectively, was denatured with 4× Laemmli SDS loading buffer and incubated for 10 min at room temperature (note: 80°C caused membrane proteins to crash out, so room temperature incubation was essential). Samples were loaded into a Novex wedgewell 10% Tris-Glycine Mini Gel (Thermo Fisher Scientific Cat. No. XP00105BOX) along with a 1:10 diluted PageRuler Plus Prestained Protein Ladder (Thermo Fisher Scientific Cat. No. 26619). Gels were run at 225 V for 30 min, and afterward, the gel was transferred to a 0.45 µM nitrocellulose membrane (Bio-Rad Cat. No. 1620115) at 50 V for 30 min, and then 100 V for 60 min in transfer buffer (25 mM tris base; 192 mM glycine; 10% methanol) at 4°C. Afterward, the membrane was stained with total protein stain (LI-COR Biosciences Cat. No. 926-11010) and then imaged in the 700 nm channel with a 30 second exposure on a LI-COR Odysey Fc. Afterward, the membrane was blocked in LICOR Intercept Blocking Buffer (LI-COR Biosciences Cat. No. 927-70001) for 1 h at 37°C and then overnight at 4°C. The following day, the membrane was immunostained with FluoTag-X2 anti-ALFA LI-COR IRDye 800CW (NanoTag Biotechnologies Cat. No. N1502-Li800-L) diluted 1:10,000 in LI-COR PBS block plus 0.2% Tween-20 for 2 h at room temperature. (Note: both the LI-COR PBS block and anti-ALFA IRDye 800CW were essential for visualization; other blocks and fluorophores lead to significantly lower signal.) The membrane was washed 3× in PBST (PBS with 0.2% Tween-20) and then imaged in the 800 nm channel with a 10 min exposure. This western protocol was adapted from a previous study (115). The protocol link is https://dx.doi.org/10.17504/protocols.io.3byl4987zgo5/v1.

### Iron acquisition homolog identification and tree construction

We compiled a list of iron acquisition proteins from *Homo sapiens* (59) to search for in the *S. rosetta* genome. Putative homologs were identified through BLAST. For phylogenies, we retrieved the corresponding Pfam hidden Markov model (HMM) from Interpro (https://www.ebi.ac.uk/interpro/) (123). Using HMMER v3.4 (hmmer.org) (124), we searched proteomes from EukProt v3 (125), focusing on proteomes from “The Comparative Set” (TCS), which consisted of 196 species of phylogenetic relevance with high BUSCO completion. We supplemented the TCS with the transcriptomes of 18 choanoflagellate species (46) and other Holozoa and Fungi. To count the number of unique proteins that conformed to the HMM profile from “hmmsearch,” we aligned hits with “hmmalign” and retained only one sequence from hits with >99% identity (note: this was important for removing multiple isoforms and reference sequences from highly annotated genomes). We additionally filtered hits based on length with “esl-alimanip” to retain protein sequences that only possessed the HMM profile, which eliminated multidomain proteins that only had the HMM profile as one of its domains. We aligned sequences with MAFFT-DASH (69), removed sites that were uninformative or contained 90% gaps with ClipKIT (126) (kpic-gappy 0.9), and generated phylogenetic trees with IQ-TREE (127) using ModelFinder (128) to choose the appropriate substitution model for each set of protein alignments. This procedure was used to determine the phylogenetic relationships of Cytb561 paralogs (Fig. 4A), and the chosen substitution model was LG + F + R7. Trees were visualized with iTOL (129). Multiple sequence alignments were visually inspected and curated for supplemental figures using Jalview v2 (130). Cytb561 structure models were predicted with AlphaFold v3 (74) and visualized using UCSF ChimeraX (73).

### Gene distribution maps

#### Geographic detection of ASVs congeneric to *S. rosetta*

The identification of *S. rosetta* was determined using metaPR2 (88), a database of 18S rRNA metabarcodes. Using only the Tara Oceans V9 data set, we searched for *S. rosetta* and close relatives (Obazoa > Opithsokonts > Choanoflagellata > Choanoflagellatea > Choanoflagellatea_X > Choanoflagellatea_XX > Salpingoeca > Salpingoeca_rosetta). Locations where ASVs annotated as *S. rosetta* were collected and used for mapping. ASVs were aligned with mafft (69) and visualized with JalView (130).

#### Geographic Cytb561 homolog search

We searched for the abundance and location of choanoflagellate Cytb561 paralogs in MATOUv1 +G (for metagenomes) and MATOUv1 +T (for metatranscriptomes) databases through the Ocean Gene Atlas (85, 86). First, we performed a BLAST search (tblastn) using *S. rosetta* paralogs as the initial query with the following parameters: expected threshold E-values < 1^−10^, and abundance as percent of total reads. The resulting E-values table was downloaded for the unique IDs for each hit, and protein sequences were pulled for the corresponding IDs. The protein sequences were then placed into a tree by using the “Constrained Tree Search (-g)” option in IQ-TREE with the tree built for Fig. 4A as the initial input. Paralogs were assigned to Group A, Group B, or Group C based on the clade placement of the resulting tree. After establishing the phylogenetic placement, the unique identifiers from the MATOU data set were used to search the Ocean Gene Atlas with the same search criteria as above. Finally, the abundance matrix and environmental parameters tables were downloaded from the search results in Ocean Gene Atlas. From these tables, the abundance, sample sites, and site coordinates were pulled and mapped.

#### Map plotting

Maps were plotted with paralog abundances and coordinates, using matplotlib with modified scripts generated by ChatGPT (https://chat.openai.com).

## Data Availability

RNA sequencing data are available in the public repository NCBI Gene Expression Omnibus (GEO) database under requisition ID GSE267344.

## SUPPLEMENTAL MATERIAL

Supplemental material msphere.00917-24-s0001.pdf Supplemental figures and descriptions of supplemental tables and movies.

Table S1 msphere.00917-24-s0002.xlsx RNAseq metadata.

Table S2 msphere.00917-24-s0003.xlsx Recipes for media.

Table S3 msphere.00917-24-s0004.xlsx Oligonucleotides.

Table S4 msphere.00917-24-s0005.xlsx Strains.

Movie S1 msphere.00917-24-s0006.mp4 Thecates phagocytosing ferric colloids.

Movie S2 msphere.00917-24-s0007.mp4 Seasonal abundance of small particulate iron taken from the PISCES model.

Movie S3 msphere.00917-24-s0008.mp4 Seasonal abundance of iron particles between 0.2 and 35 μm taken from the PISCES model.

ASM does not own the copyrights to Supplemental Material that may be linked to, or accessed through, an article. The authors have granted ASM a non-exclusive, world-wide license to publish the Supplemental Material files. Please contact the corresponding author directly for reuse.
