# Supplementary material for: Cell differentiation controls iron assimilation in the choanoflagellate Salpingoeca rosetta
Source: mSphere. 2025 Feb 26;10(3):e00917-24. doi: 10.1128/msphere.00917-24 (PMC11934334; doi:10.1128/msphere.00917-24)
Supplement: Supplemental material — Supplemental figures and descriptions of supplemental tables and movies. [file msphere.00917-24-s0001.pdf]

Supplementary materials for

Cell differentiation controls iron assimilation in the choanoflagellate *Salpingoeca rosetta*

Authors:

Fredrick Leon[1,3], Jesus M. Espinoza-Esparza[1], Vicki Deng[1,5], Maxwell C. Coyle[4,6], Sarah Espinoza[4], David S. Booth[1,2*]

Affiliations:

[1]Department of Biochemistry and Biophysics
University of California, San Francisco School of Medicine
San Francisco, CA

[2]Chan Zuckerberg Biohub SF
San Francisco, CA

[3]Tetrad Graduate Program
University of California, San Francisco
San Francisco, CA

[4]Howard Hughes Medical Institute &
Department of Molecular and Cell Biology
University of California, Berkeley
Berkeley, CA

[5]Current Address: Department of Molecular Biosciences
University of Texas, Austin
Austin, TX

[6]Current Address: Department of Molecular and Cellular Biology
Harvard University
Cambridge, MA

[*]Corresponding author: David.Booth@ucsf.edu

Table of Contents

Section	Page(s)
Supplementary Figures	3-14
S1: A preferential lysis buffer enriches RNA from *S. rosetta* to improve transcriptomes.	3
S2: GO molecular functions and biological processes enriched in thecates and slow swimmers.	5
S3: *S. rosetta* cell types, but not their feeder bacteria, grow differently with variable iron sources.	7
S4: Thecates phagocytose ferric colloids.	9
S5: A screen for optimal nucleofection pulses to improve cargo delivery into *S. rosetta*.	11
S6: Genotypes of engineered strains.	13
S7: Validated qPCR primers confirm the disruption of *cytb561a* expression with the *cytb561a*^{PTS} allele.	15
S8: Thecates grow slightly faster and to a larger population size with ferric colloids.	17
S9: Cytb561a is not detectable in slow swimmers.	19
S10: Cytochrome b561 paralogs have retained ascorbate binding residues.	21
S11: Cytochrome b561 dimerization is a feature of Group A proteins.	23
S12: Cytb561a is predicted to dimerize similarly to human DCYTB.	25
S13: The lumenal surfaces of Cytochrome b561 feature electrostatic properties that are general features of Cytochrome b561 subgroups.	27
S14: Phylogenetic and geographic distribution of Cytb561 homologs congeneric to *S. rosetta*	29
Supplementary Tables	31
Table S1: RNA sequencing metadata.	See attached .xlsx files
Table S2: Media table.	
Table S3: Table of oligos and gRNAs for mutant generation and screening.	
Table S4: Strain table.	
Supplementary Movies	32
Movie S1: Thecates phagocytose ferric colloids.	See attached .mp4 files
Movie S2: Congeneric ASVs and Cytb561a paralogs found in regions of the ocean with dissolved iron.	
Movie S3: Congeneric ASVs and Cytb561a paralogs found in regions of the ocean with small particulate iron.	

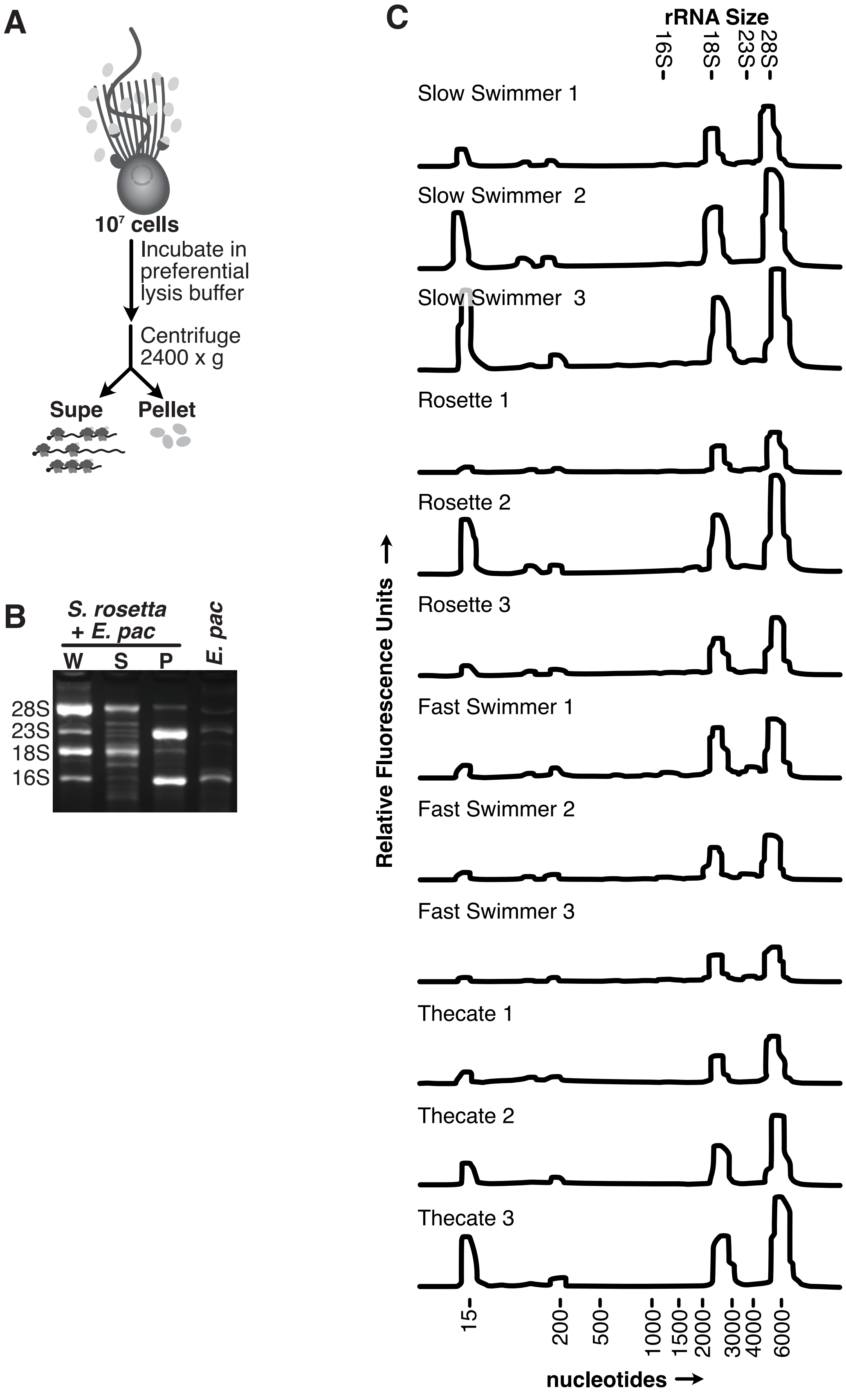

Figure S1. A preferential lysis buffer enriches RNA from *S. rosetta* to improve transcriptomes.

Figure S1. A preferential lysis buffer enriches RNA from *S. rosetta* to improve transcriptomes.
(A) A schematic showing the separation of *S. rosetta* RNA from *E. pacifica* RNA.
(B) The preferential lysis procedure enriches RNA from *S. rosetta*. After performing preferential lysis, RNA was purified from the supernatant using Trizol LS reagent. The RNA from the pellet of preferential lysis, an unlysed sample of *S. rosetta* feeding on *E. pacifica*, or a sample of *E. pacifica* was purified with Trizol LS. The purified RNA samples were combined with an equal volume of formamide and incubated at 95°C for 5 minutes. After cooling to room temperature, the samples were run on a 2% (w/v) agarose gel in TBE buffer at 100 V. Afterwards, the gel was stained with SYBR Gold to detect RNA. In the gel, the supernatant (S) from whole lysates (W) mostly contains *S. rosetta* rRNA (28S and 18S); the pellet (P) contains E. pacifica rRNA (23S and 16S).
(C) Total RNA prepared for RNA-seq primarily contains rRNA from *S. rosetta.* Bioanalyzer traces with predicted prokaryotic and eukaryotic rRNA subunit sizes indicated. Replicate RNA samples used for sequencing demonstrate little to no carryover from bacterial contamination, as shown by the low amount of 16S and 23S rRNA.

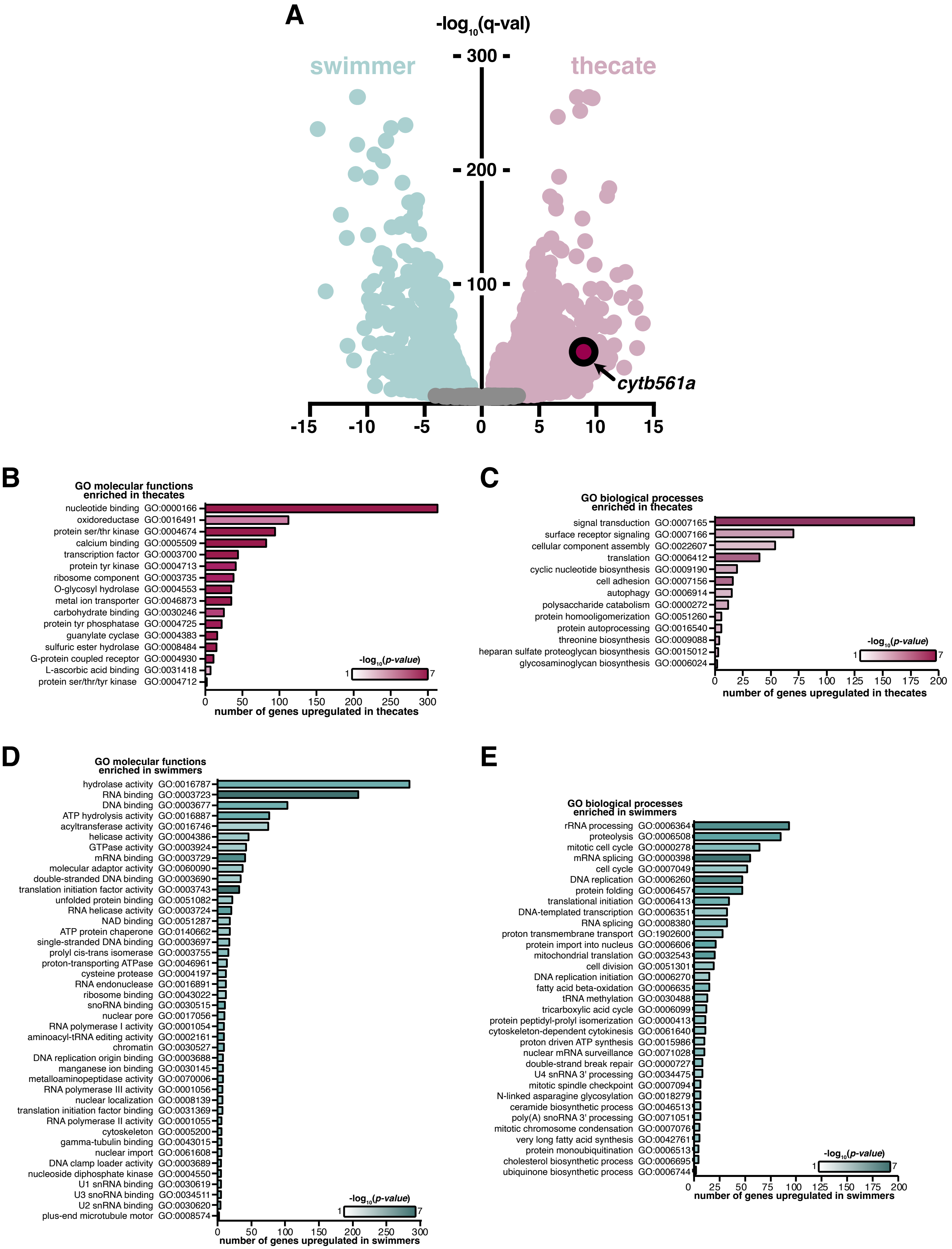

Figure S2: GO molecular functions and biological processes enriched in thecates and slow swimmers.

Figure S2: GO molecular functions and biological processes enriched in thecates and slow swimmers.

(A) *cytb561a* is highly upregulated in the thecate cell type. Volcano plot shows the distribution of upregulated thecate and slow swimmer genes, with *cytb561a* denoted by the circle.

(B, C) Functional modules enriched in thecates mediate signal transduction, nutrient acquisition, and gene regulation. Genes with transcript abundances that reliably ($q < 0.01$) changed more than two-fold in thecates were analyzed for the enrichment of Gene Ontology (GO) categories, focusing on GO molecular functions **(B)** and biological processes **(C)**.

(D, E) Functional modules enriched in swimmers support RNA processing, mitosis, and metabolism. Genes with transcript abundances that reliably ($q < 0.01$) changed more than two-fold in swimmers were analyzed for the enrichment of Gene Ontology (GO) categories, for GO molecular functions **(D)** and biological processes **(E)**. Genes that were upregulated and associated with an enriched GO molecular function category were counted (x-axis). Each bar on the graph is colored by the *P*-value, which reflects the probability that a given category was represented by a random selection of genes from the *S. rosetta* genome.

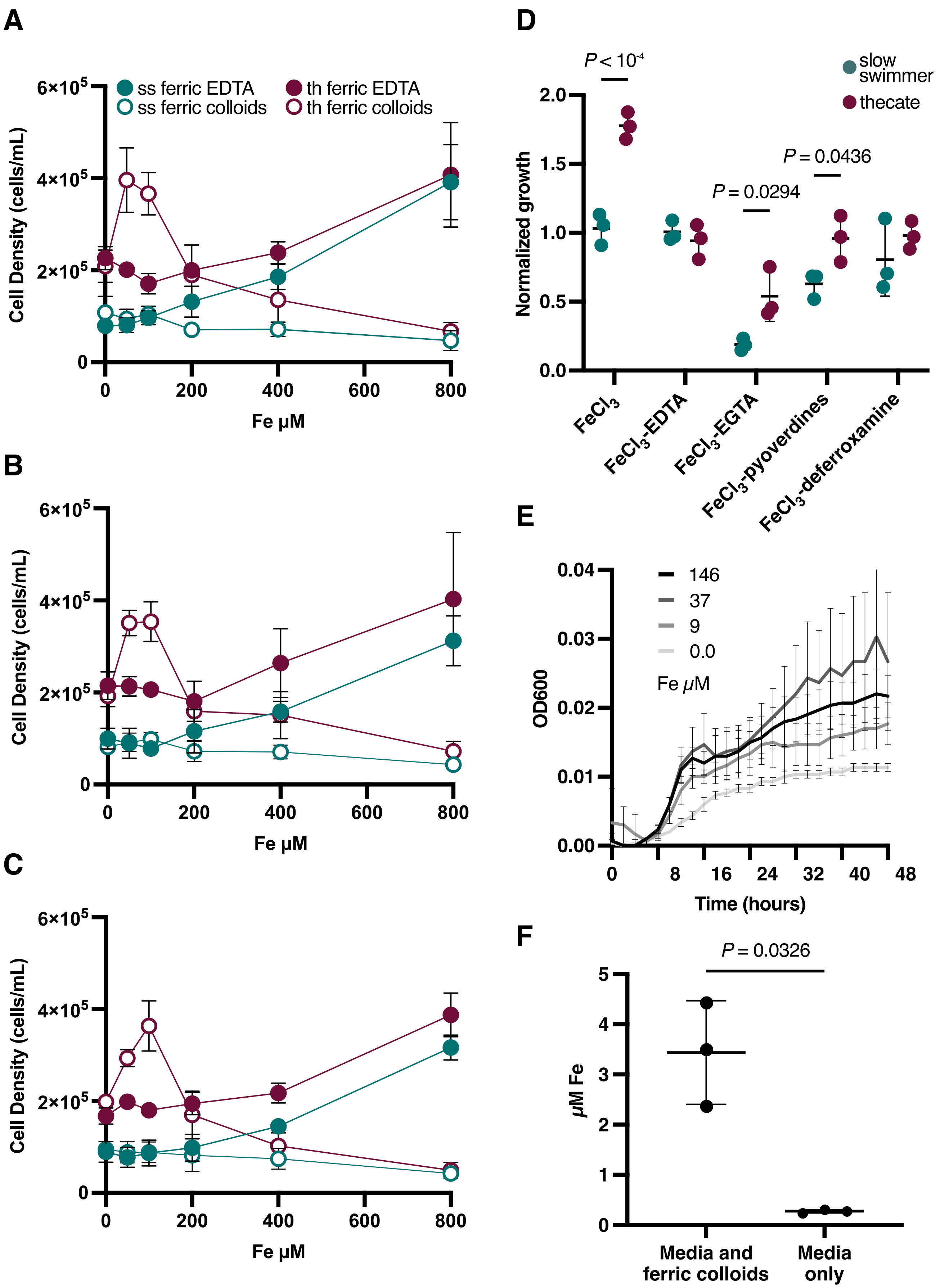

Figure S3: ***S. rosetta*** **cell types, but not their feeder bacteria, grow differently with variable iron sources.**

Figure S3: *S. rosetta* cell types, but not their feeder bacteria, grow differently with variable iron sources.

(A-C) Thecate cell types exhibit differential growth with low concentrations of ferric colloids compared to slow swimming cell types. *S. rosetta* cell density of slow swimmer and thecate cultures when grown with titrations of ferric EDTA or ferric colloids separated by independent tricplicates. Data shown in Fig. 2B shows the average of each triplicate shown here.

(D) A variety of ferric chelates differentially impact *S. rosetta* growth. Slow swimmers and thecates exhibit different growth characteristics, so to better compare their growth with iron bound to different chelators, we used a ratio of the final cell density of cultures grown with iron-chelator complexes to the final cell density of cultures grown without any supplemental iron. With this metric, a ratio greater than one indicates that the cell type displays increased growth with the iron-chelator complex; whereas, a ratio less than one indicates the converse. All conditions were tested at 100 µM Fe^{3+}, and pyoverdines and deferroxamine are bacterial siderophores.

(E) *E. pacifica* feeder bacteria growth is easily rescued by low concentrations of iron. Optical density at 600 nm (OD600) of *E. pacifica* cultures grown with titrations of ferric EDTA measured approximately every 100 minutes for 48 hours. Note standard deviations overlap for all conditions except for a no iron control.

(F) Ferric colloids release a small amount of labile iron. When media is prepared with 100 µM ferric colloids, only 3.43 ± 1.01 µM iron is liberated without biological activity. The amount of iron in 4% PG media alone was 0.27 ± 0.036 µM iron.

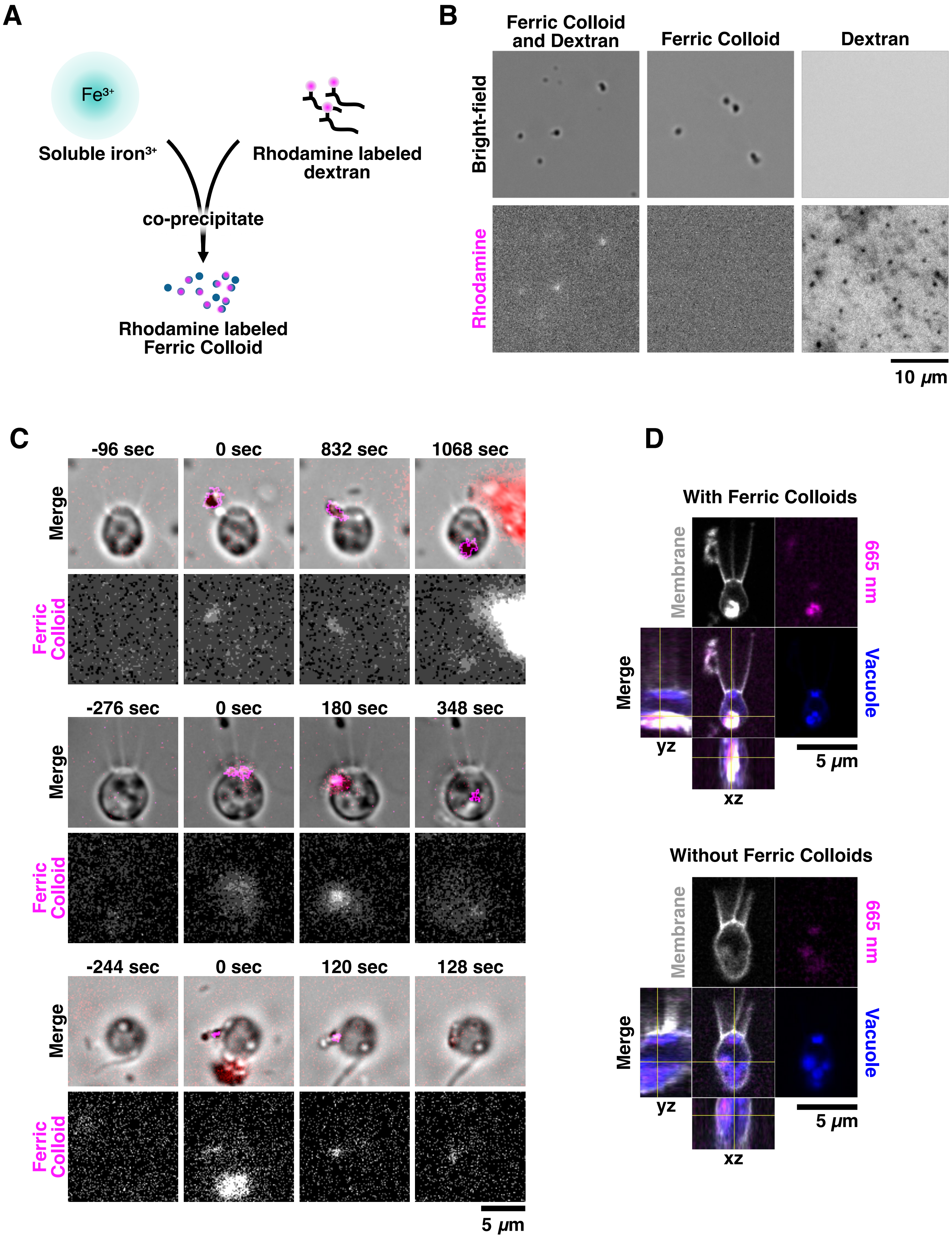

Figure S4: Thecates phagocytose ferric colloids.

Figure S4: Thecates phagocytose ferric colloids.

(A) Fluorescently labeled ferric colloids were embedded with fluorescent dextrans. During the precipitation of $FeCl_3$ to make ferric colloids, fluorescently labeled dextrans were added. The resulting colloids were incubated with dextranase, washed, and then resuspended in ASW before adding to thecate cultures for time-course microscopy.

(B) Embedded fluorescent dextrans highlight particles of ferric colloids. The images show colloids of similar size and density, with only the co-precipitated ferric colloids and dextran exhibiting fluorescence.

(C) Corroborating examples of time-lapse ferric colloid ingestion. Time courses of thecates feeding on ferric colloid particles (magenta) are displayed in the fluorescence channel (below) and in a merge with brightfield and fluorescence channel. 0 seconds denotes the initial point of contact between the cell and the tracked colloid particle.

(D) Corroborating examples of confocal ferric colloid internalization. Confocal microscopy of thecates fed with and without fluorescently labeled ferric colloids. 665 nm denotes the channel for ferric colloid detection, and orthogonal views are presented of the merged channel.

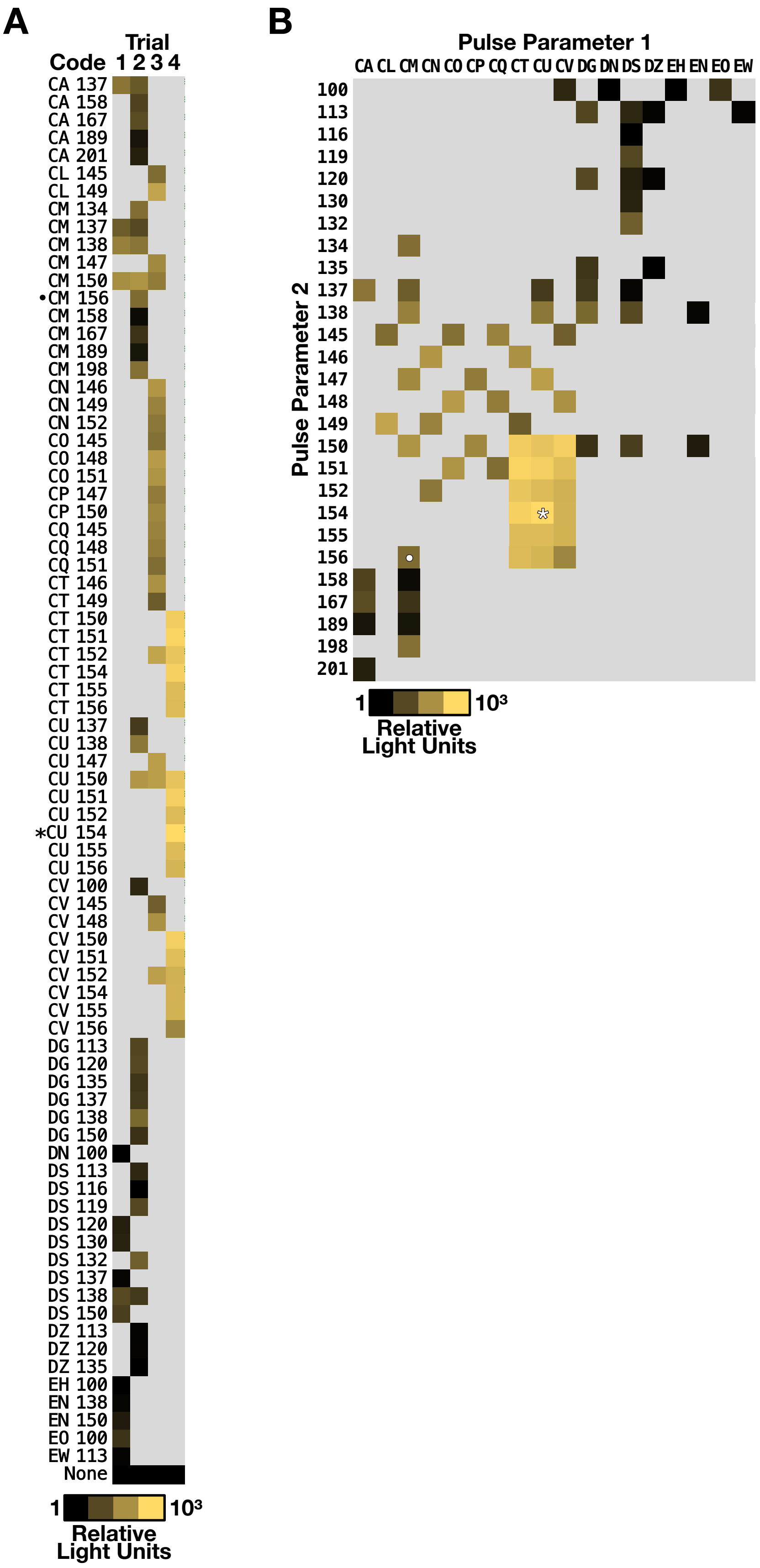

Figure S5: A screen for optimal nucleofection pulses to improve cargo delivery into *S. rosetta*.

Figure S5: A screen for optimal nucleofection pulses to improve cargo delivery into *S. rosetta*.
(A) Nanoluc luminescence values from *S. rosetta* cultures transfected with different pulse codes. Because of changes to the growth media for *S. rosetta*, we conducted a new screen for an optimal nucleofection pulse. Cells were transfected with a plasmid that drives nanoluc expression with a promoter cloned from elongation factor L. Pulse codes correspond to Lonza 4D-Nucleofector codes. The screen was performed in four independent trials. Dot indicates prior pulse code used, asterisk denotes optimized pulse code.
(B) Re-ordered chart of luciferase activity from the pulse screen reveals the landscape to optimize nucleofection pulses. After the first two trials for screening pulses, we hypothesized that the letter and number codes for the nucleofection system are orthogonal parameters, so we selected pulses for the third and fourth trials to more broadly sample the optimization landscape. The maximum luminescence value for each pulse from one of the four trials was used to visualize transfection efficiency as a function of the letter codes (x-axis) or pulse numbers (y-axis). Dot indicates prior pulse code used, asterisk denotes optimized pulse code.

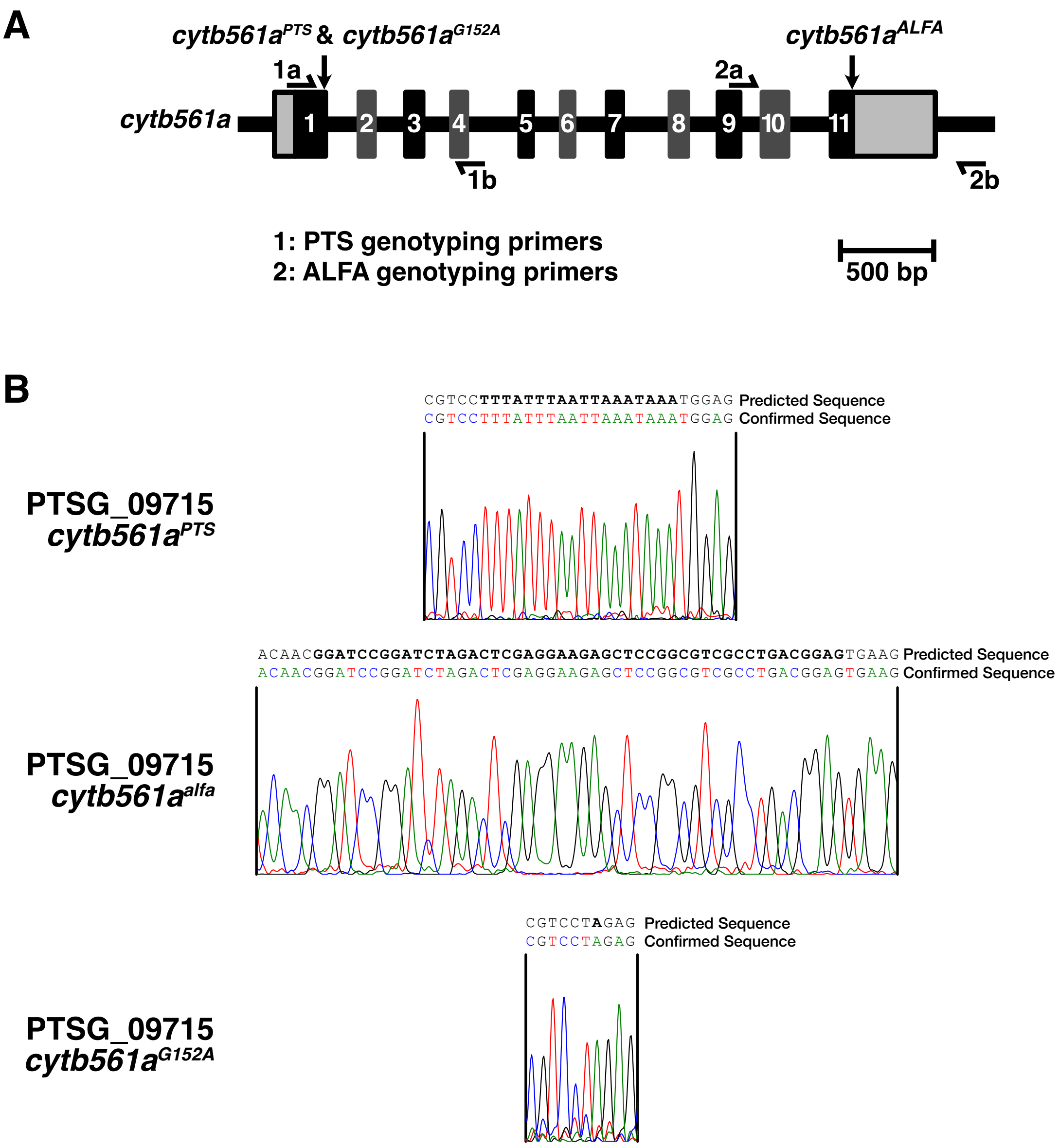

Figure S6: Genotypes of engineered strains.

Figure S6: Genotypes of engineered strains.

(A) Diagram of the *cytb561a* locus showing the location of edits and the respective genotyping primers.

(B) For each strain that was engineered, the targeted locus (allele names shown to the left) was amplified by PCR and then sequenced with the Sanger method. The sequencing traces for locus are displayed with the predicted and confirmed sequences.

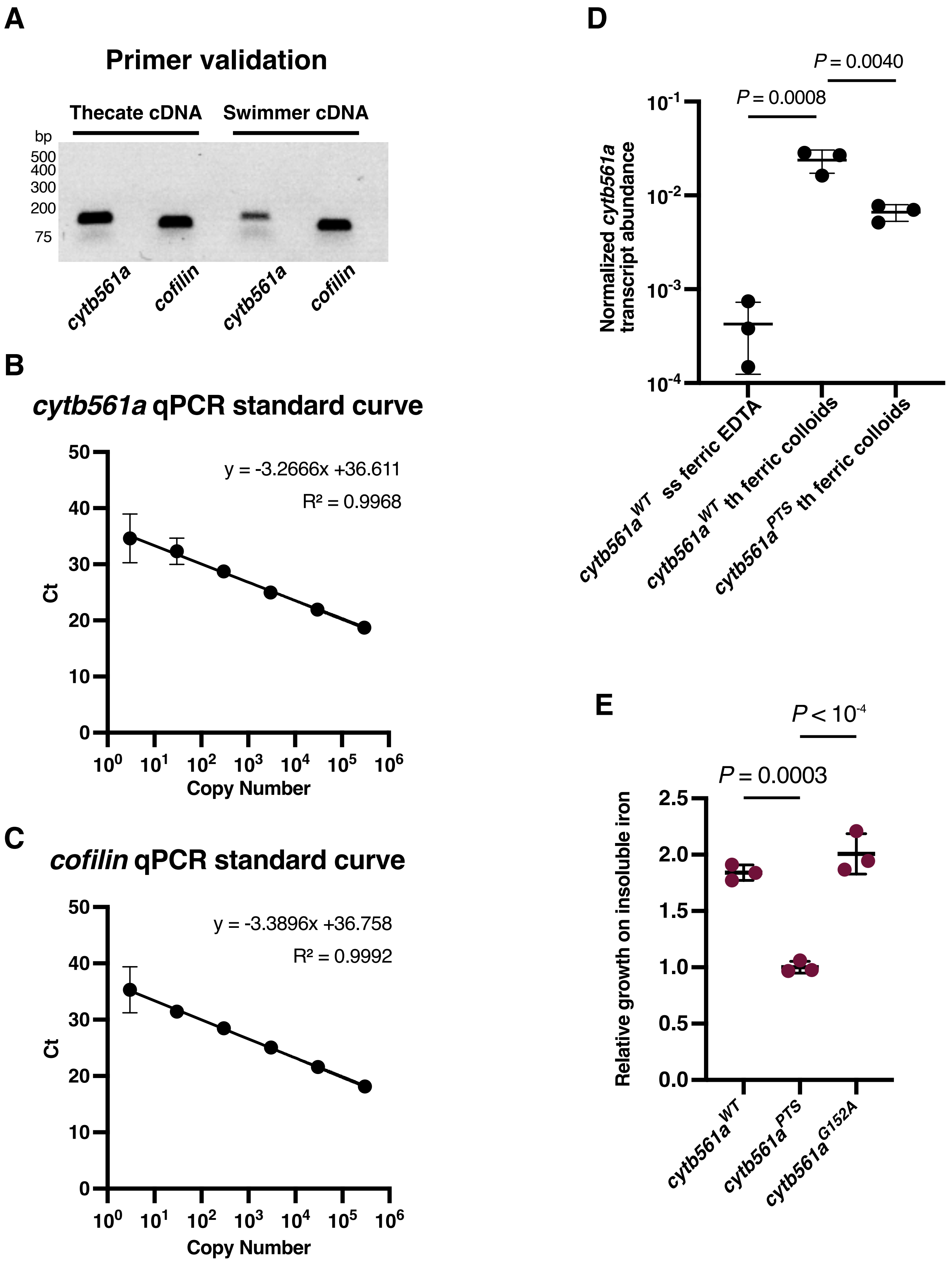

Figure S7: Validated qPCR primers confirm the disruption of *cytb561a* expression with the *cytb561a*^{PTS} allele.

Figure S7: Validated qPCR primers confirm the disruption of *cytb561a* expression with the *cytb561a*^{PTS} allele.

(A) qPCR primers for *cytb561a* and *cofilin* produce a single amplicon. Gel image of PCR products amplified from qPCR primers designed for *cytb561a* and *cofilin*. Note the diminished band from swimmer cDNA likely resulted from lower mRNA copies from slow swimmer cultures, as shown in Fig. 2C. Shadow at 75 bp is a shadow of the loading dye.

(B) Standard curve for qPCR primers for *cytb561a*. To generate a standard curve, a serial dilution of ssDNA standards was performed in triplicate. The concentration of the ssDNA standard was determined by Qubit. Cycle thresholds (Ct) were converted to transcript copy number by fitting a linear equation to the standard curve. This curve was used to determine the abundance of transcripts from three independent experiments.

(C) Standard curve for qPCR primers for *cofilin*. The graph is laid out as for panel B.

(D) *cytb561a*^{PTS} exhibits lowered *cytb561a* expression compared to wild-type thecates. We compared the normalized expression of *cytb561a* from the strain bearing the *cytb561a*^{PTS} allele to wild-type strains. The wild-type strains were slow swimmers cultured with ferric EDTA or thecates cultured with ferric colloids, for we anticipated that these two conditions would result in the greatest difference in *cytb561a* expression.

(E) Reversion of *cytb561a*^{PTS} allele restores the growth advantage of thecates fed on ferric colloids. The cell density of thecate cultures grown with ferric colloids was normalized to cultures grown with ferric EDTA. With this metric, a ratio greater than one indicates that the cell type displays increased growth with ferric colloids, whereas a ratio less than one indicates the converse. The premature termination sequence introduced at position 151 in *cytb561a*^{PTS} was reverted to the wild-type protein sequence with the single synonymous mutation G152A to distinguish it from wild type, creating the allele *cytb561a*^{G152A}.

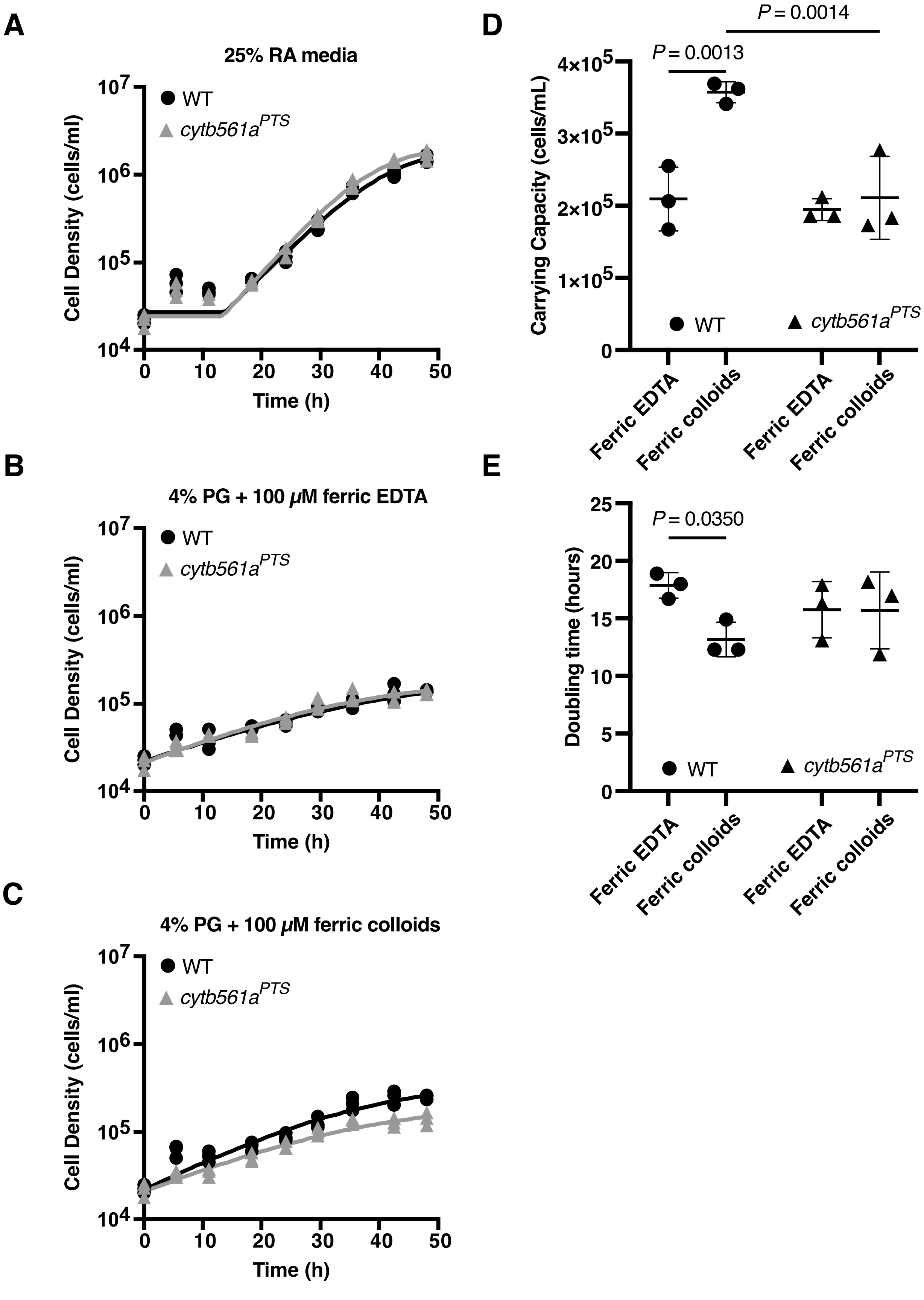

Figure S8: Thecates grow slightly faster and to a larger population size with ferric colloids.

Figure S8: Thecates grow slightly faster and to a larger population size with ferric colloids.
(A-C) Growth curves of wild type and *cytb561a*^{PTS} thecates show wild type thecates grow to a larger population size when grown with ferric colloids. Growth curves of cultures grown in (A) nutrient replete conditions (25% RA), (B) 4% PG with ferric EDTA, and (C) 4% PG with ferric colloids. Cell densities were counted every 6 hours for 48 hours.
(D-E) Comparisons of growth curve parameters highlight the increase in carrying capacity as the main factor for higher wild type thecate cell type growth with ferric colloids. (D) Carrying capacity, and (E) doubling time of the growth curves shown in figures (B) and (C).

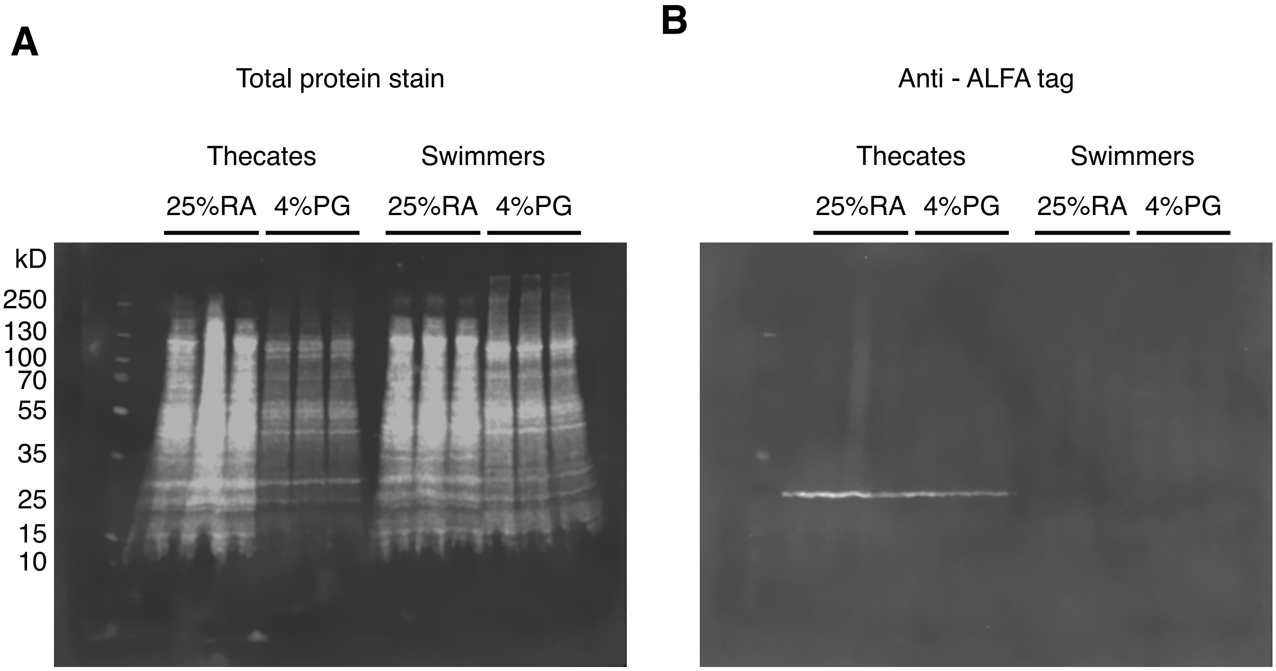

Figure S9: Cytb561a is not detectable in slow swimmers.

Figure S9: Cytb561a is not detectable in slow swimmers.

Western blot of *cytb561a*^{ALFA} thecates and swimmers grown in nutrient rich media (25% RA) and iron depleted media (4% PG) show a slight change in Cytb561a levels in response to iron availability, but no detectable amounts of Cytb561a in slow swimmers from either media condition.

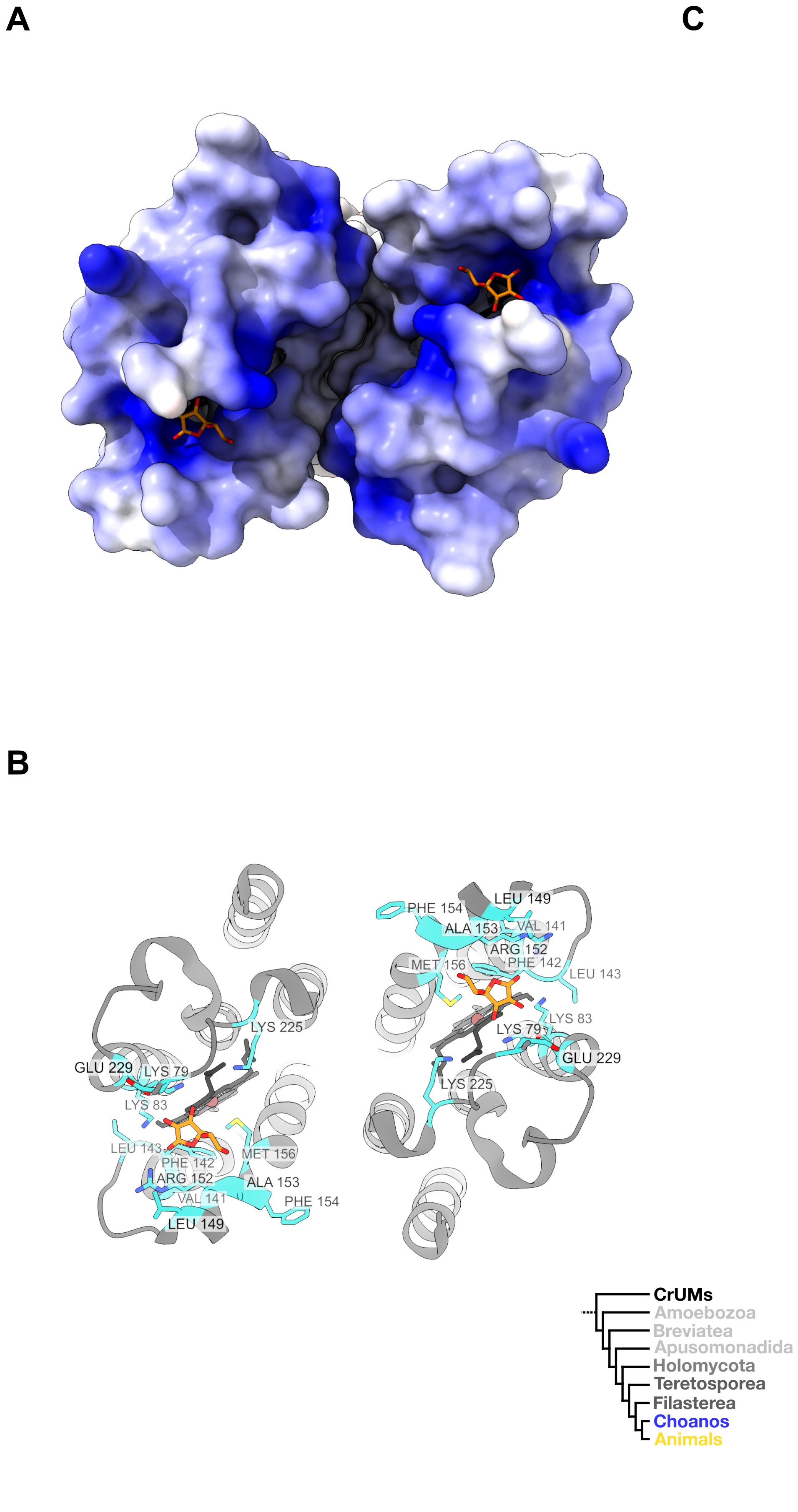

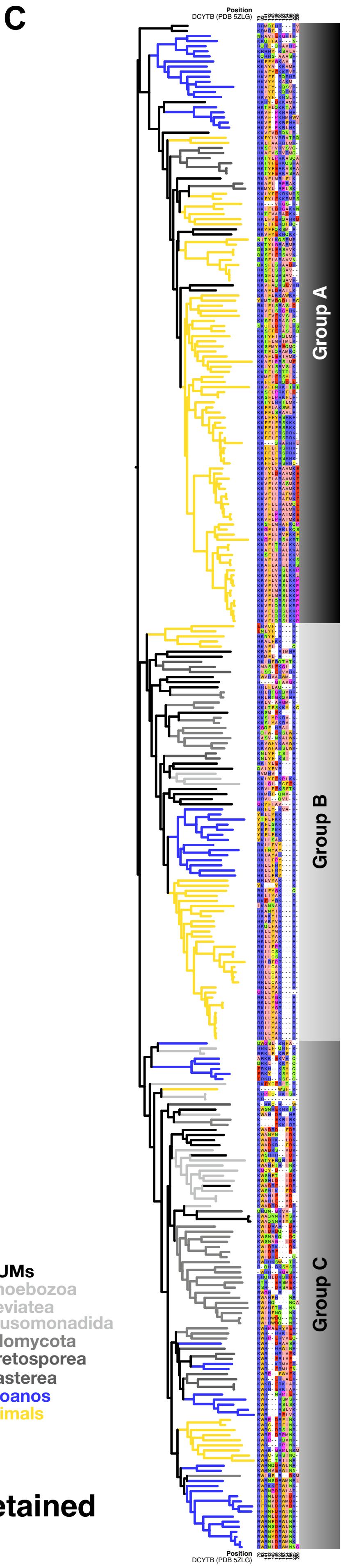

Figure S10: Cytochrome b561 paralogs have retained ascorbate binding residues.

Figure S10: Cytochrome b561 paralogs have retained ascorbate binding residues.
(A-B) The ascorbate binding pocket of human cytochrome b561 protein DCYTB. The crystal structure (PDB 5ZLG) of human duodenal cytochrome b561 (DCYTB) features a positively charged binding pocket on the cytosolic face of each polypeptide of the homodimeric complex **(A)**. Each pocket binds one ascorbate molecule (orange) that is coordinated by specific electrostatic and hydrophobic contacts **(B**, cyan residues**)**
(C) Cytb561 homologs have retained ascorbate binding residues. An alignment of cytochrome B561 protein sequences shows conservation in positions that correspond to ascorbate-binding residues in human DCYTB.

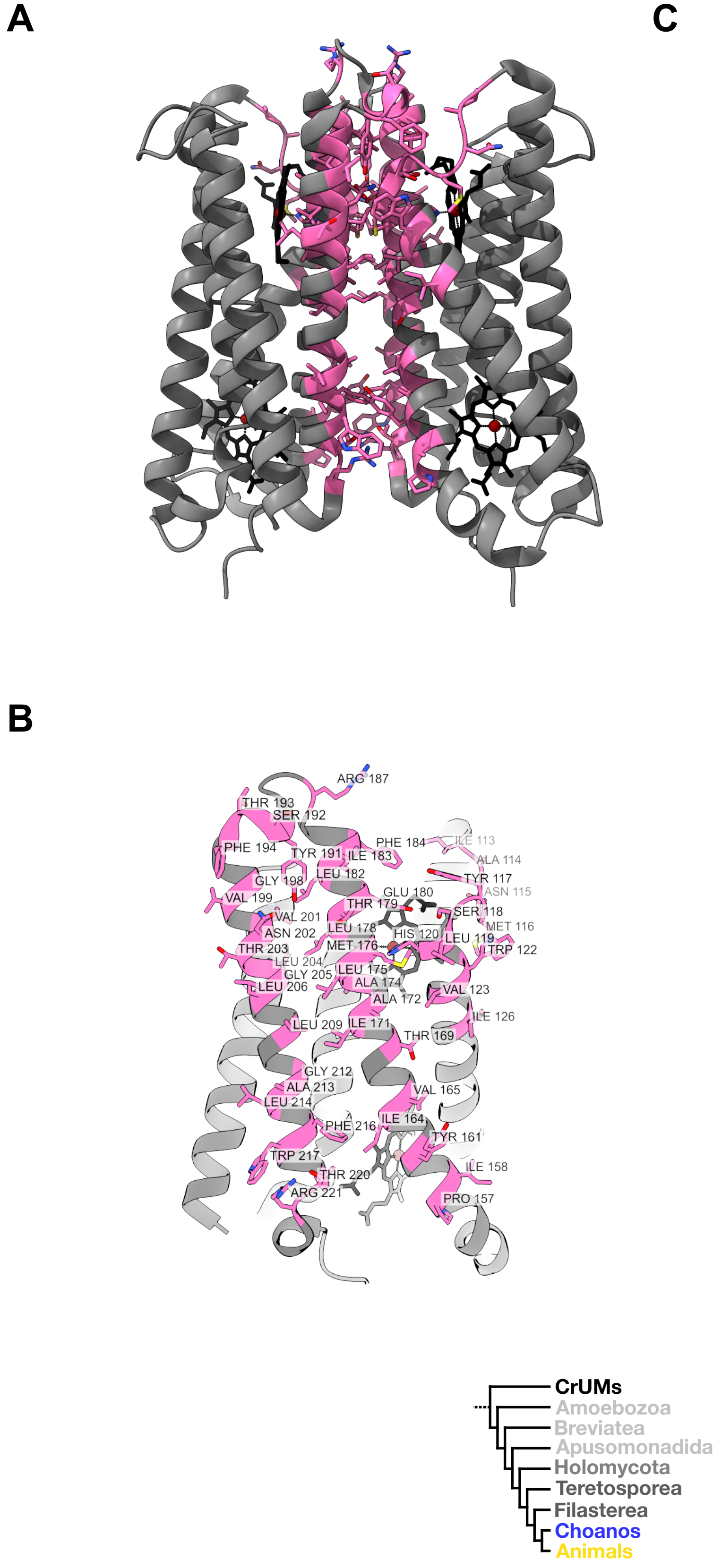

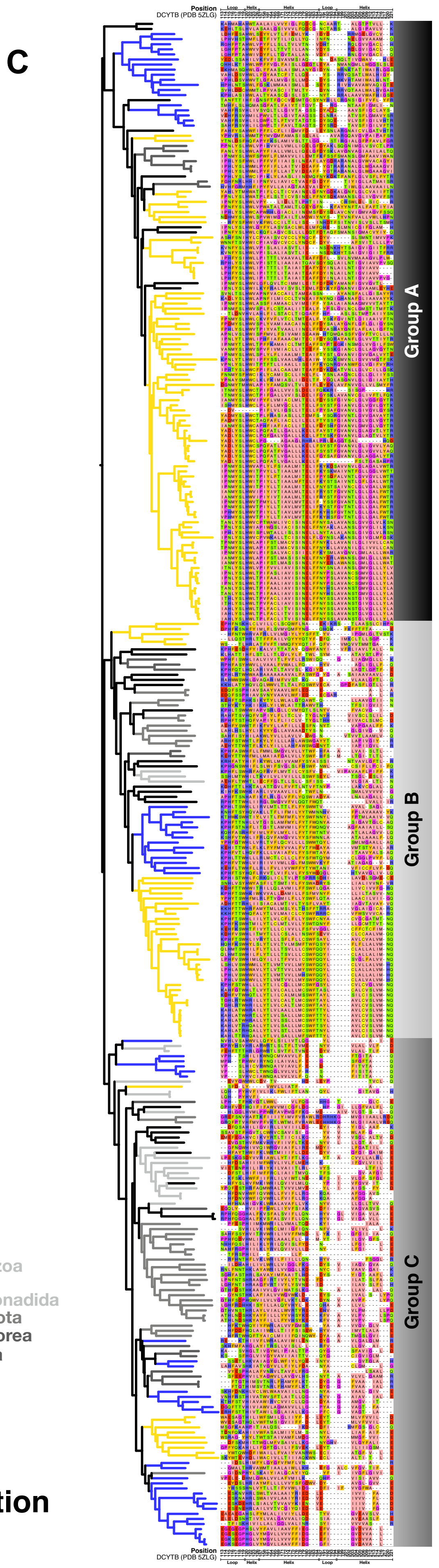

Figure S11: Cytochrome b561 dimerization is a feature of Group A proteins.

Figure S11: Cytochrome b561 dimerization is a feature of Group A proteins.

(A-B) The crystal structure of human DCYTB (PDB 5ZLG) forms a homodimeric complex **(A)**. Residues that mediate homodimerization **(B, pink residues)**. Heme groups are depicted in black with coordinated iron atoms in red.

(C) Cytochrome b561 homologs from Group A, including human DCYTB and *S. rosetta* Cytb561a, have key residues for dimerization, while groups B and C do not. An alignment of cytochrome b561 protein sequences reveals that Group A homologs conserve similar residues at positions that correspond to the dimerization residues in DCYTB. Sequences in Group B do not align at ~7 positions corresponding to the dimeric interface, and Group C sequences do not align at ~10 positions.

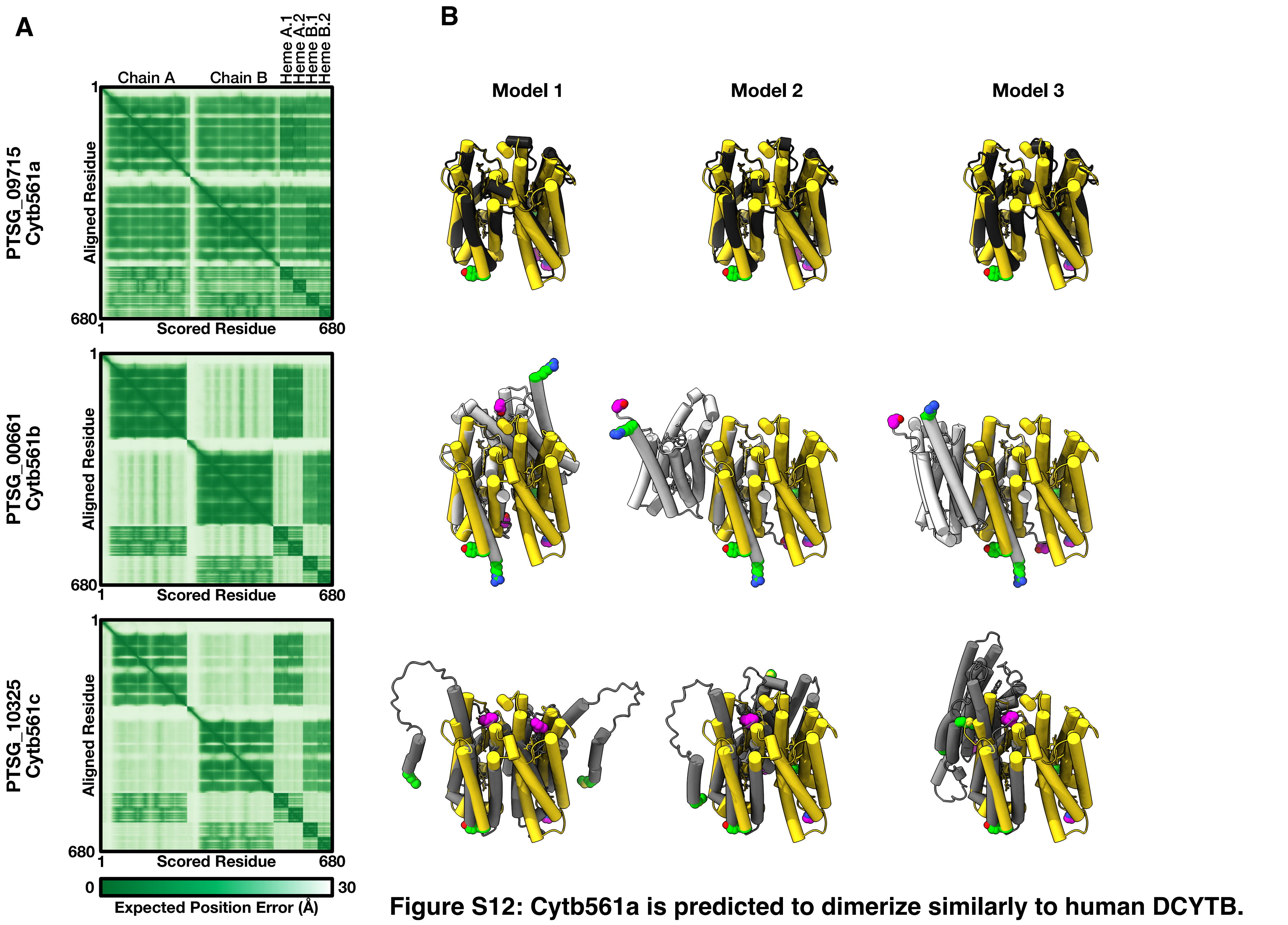

Figure S12: Cytb561a is predicted to dimerize similarly to human DCYTB.

Figure S12: Cytb561a is predicted to dimerize similarly to human DCYTB.

(A) Expected position errors of dimerization predictions indicate that Cytb561a robustly forms a dimeric complex. Dimeric structures of each Cytb561 paralog bound to heme cofactors were predicted with AlphaFold v3. The expected position error graphs from those predictions depict the distance between the same residue across predicted dimer models. The smaller the expected distance between residues indicates greater consistency between different models generated during the prediction.

(B) Predicted dimers of Cytb561a consistently align with human DCYTB dimers. The top three predicted models of Cytb561 paralogs from *S. rosetta* (grey structures) were aligned to the crystal structure of human DCYTB. Alignments were restricted to only one chain from each dimeric complex, allowing the second chain to be positioned based on the predicted structure. To better visualize the position of each dimer, the amino-terminal residue is colored in green and the carboxy-terminal residue in magenta. In these alignments, all three models of Cytb561a from *S. rosetta* consistently align with the DYCTB dimer. In contrast, the second polypeptide chain from Cytb561b and Cytb561c display variable positions across all three models.

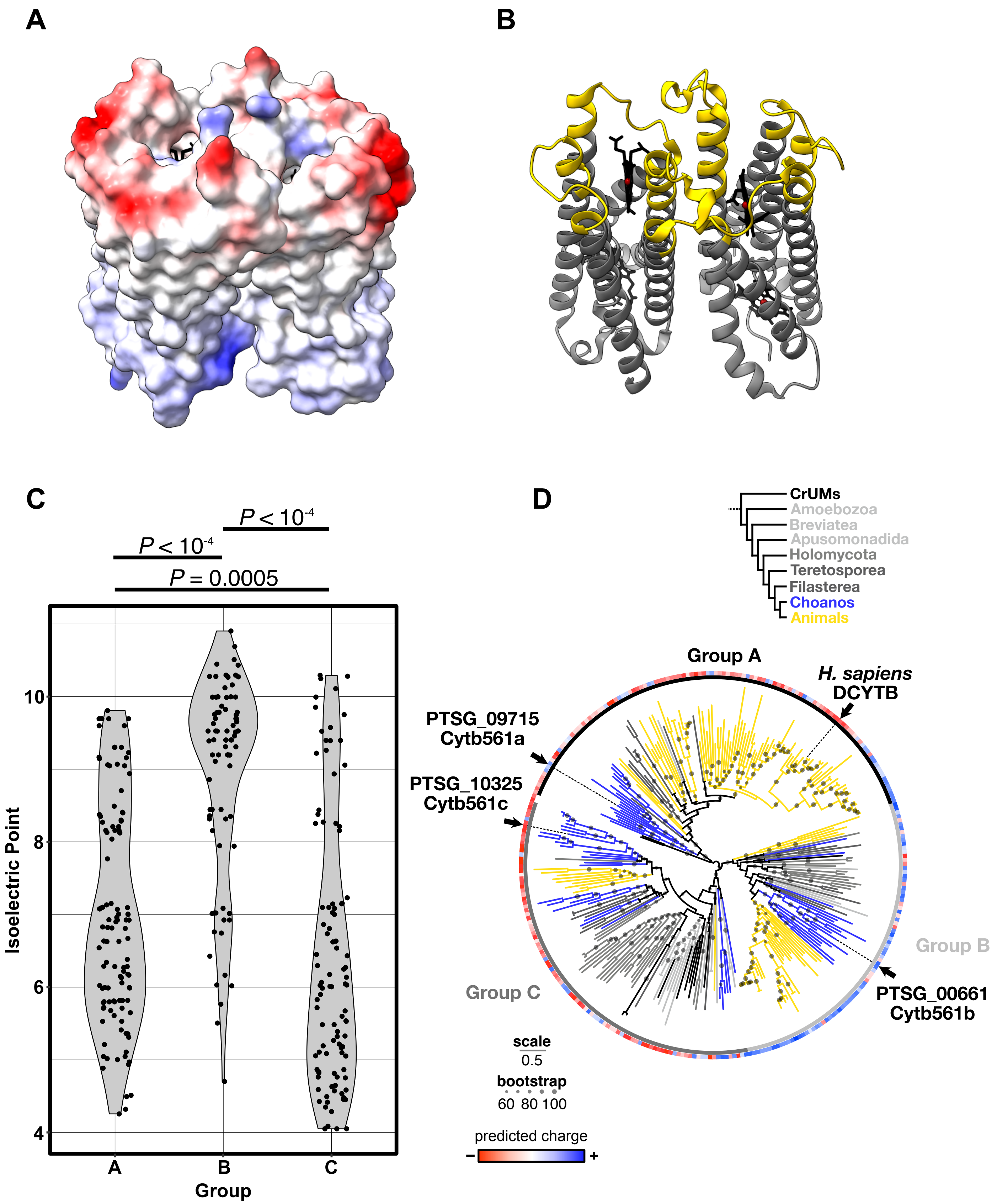

Figure S13: The lumenal surfaces of Cytochrome b561 feature electrostatic properties that are general features of Cytochrome b561 subgroups.

Figure S13: The lumenal surfaces of Cytochrome b561 feature electrostatic properties that are general features of Cytochrome b561 subgroups.

(A) The lumenal surface of the DCYTB homodimer (PDB 5ZLG) has negatively and positively charged patches that bind iron by itself or iron complexed with ligands.

(B) Loops (yellow) that bridge transmembrane helices form the lumenal surface of DCYTB.

(C) Cytb561 subgroups have distinctive electrostatic properties on their lumenal surfaces. The sequences of loops that comprise the lumenal surface of Cytochrome b561 were concatenated to calculate the isoelectric point of the lumenal surface for each paralog. Individual paralogs from each subgroup are displayed as black dots, and the distribution of charges is shown for each subgroup as a grey violin plot. A Kolmogorov-Smirnov test compared the distributions of isoelectric points between Cytochrome b561 subgroups.

(D) A character map of lumenal surface charge suggests that electrostatic properties can evolve within Cytochrome b561 subgroups. A maximum likelihood phylogeny of cytochrome B561 proteins (as in Fig. 4A) with the isoelectric points of the lumenal surface for each protein shows that surface properties are variable even in well-resolved clades.

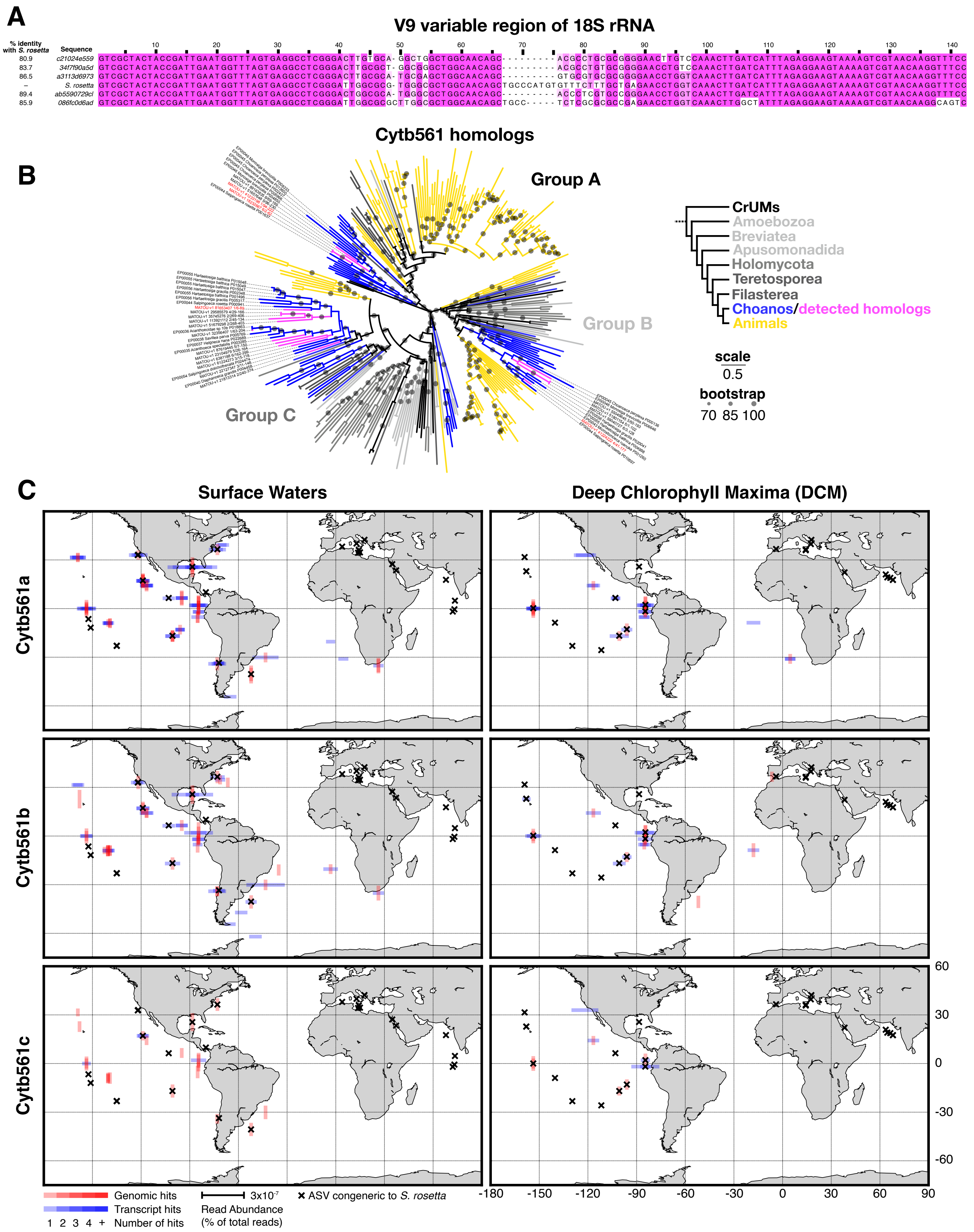

Figure S14: Phylogenetic and geographic distribution of Cytb561 homologs congeneric to *S. rosetta.*

Figure S14: Phylogenetic and geographic distribution of Cytb561 homologs congeneric to *S. rosetta*

(A) Congeners of *S. rosetta* are detectable in ocean environments. Species closely related to *S. rosetta* were identified by metaPR2, an 18S rRNA metabarcoding database. These congeneric amplified sequence variants (ASVs) were aligned to the *S. rosetta* V9 variable region of the 18S rRNA subunit. The congeneric ASVs was used to identify the geographic distribution of closely related species to *S. rosetta* (Figs. 5B).

(B) Environmental Cytb561 homologs nest within choanoflagellate clades. After searching for *S. rosetta* Cytb561 paralogs in an open repository of metatranscriptomes and metagenomic databases, closely related homologs were identified. To confirm the placement and paralog identity, the detected Cytb561 protein sequences were incorporated into the free from figure 4A. The detected paralog branches are magenta, while the unigene ID of the nearest ortholog to *S. rosetta* within each group is highlighted red.

(C) The geographic distribution of Cytb561 paralogs closely related to *S. rosetta*. Paralogs detected in an open repository of metagenomes and metatranscriptomes from marine environments were mapped. Surface waters correspond to within 5 m of the ocean surface and Deep Chlorophyll Maxima (DCM) corresponds to 20-200m from the surface, where chlorophyll levels are highest. Paralog abundance was measured as the percentage of total reads at each sample site. Red vertical lines correspond to genomic abundance, blue horizontal lines correspond to transcript abundance, and black crosses denoted sites where *S. rosetta* or near relative of *S. rosetta* amplified sequence variants (ASVs) were detected. Overlapping hits at matching locations increase the color saturation. Longitude and Latitude degrees are marked on the bottom right map.

Supplemental Tables:

Table S1: RNA sequencing metadata.
Tabulated strain IDs, culturing conditions, NCBI accession IDs, and sequencing metadata for RNA sequencing.

Table S2: Media table.
Components and recipes for all media used: 5% seawater complete (SWC0, 10% cereal grass media (CGM3), 10% red algae (RA), 25% red algae (RA), 15% red algae, 2% peptone, yeast extract, glycerol (15/2), and 4% peptone, glycerol (PG).

Table S3: Table of oligonucleotides and gRNAs for mutant generation and screening.
Tabulated guide RNAs, repair template oligonucleotides, and primers for generating, screening, and isolating mutants.

Table S4: Strain table.
The strains of *S. rosetta* used and created during this study, along with the crRNAs, repair templates, Cas12a screening gRNAs, and screening primers for generating each strain.

Movies:

Movie S1: Thecates phagocytosing ferric colloids. Four different thecates are shown eating ferric colloids. Brightfield images of cells are shown in grayscale and the fluorescent signal from rhodamine labeled ferric colloids is shown in magenta. Cell 1 is viewed from the side, oriented with the apical collar upwards and the basal end downwards. Cells 2 and 3 are oriented the same as cell 1, but for cell 3, the ingestion of the ferric colloid occurs out of the focal plane. Cell 4 is viewed from the top of the collar.

Movie S2: Congeneric ASVs and Cytb561a paralogs found in regions of the ocean with dissolved iron. The seasonal abundance of small particulate iron taken from the PISCES model shows dissolved iron and colloidal iron < 0.2 μm is present at sites where congeneric ASVs to *S. rosetta* and closely related homologs to Cytb561a are detectable.

Movie S3: Congeneric ASVs and Cytb561a paralogs found in regions of the ocean with small particulate iron. The seasonal abundance of iron particles between 0.2 – 35 μm taken from the PISCES model shows colloidal iron is present at cites where congeneric ASVs to *S. rosetta* and closely related homologs to Cytb561a are detectable.
